# Nonlinear Stochastic Dynamics of the Intermediate Dispersive Velocity Equation with Soliton Stability and Chaos

**DOI:** 10.3390/e27111176

**Published:** 2025-11-20

**Authors:** Samad Wali, Maham Munawar, Atef Abdelkader, Adil Jhangeer, Mudassar Imran

**Affiliations:** 1 General Education Centre, Quanzhou University of Information Engineering, Quanzhou 362000, China; wali_samad@qzuie.edu.cn; 2 Department of Mathematics, University of Engineering and Technology, Lahore 39161, Pakistan; mahammunawar63@gmail.com; 3 College of Humanities and Sciences, Ajman University, Ajman P.O. Box 346, United Arab Emirates; mudassar.imran@ajman.ac.ae; 4 IT4-Innovations, VSB-Technical University of Ostrava, 708 00 Ostrava-Poruba, Czech Republic; 5 Center for Theoretical Physics, Khazar University, 41 Mehseti Str., AZ1096 Baku, Azerbaijan

**Keywords:** stochastic nonlinear dynamics, Lyapunov exponents, recurrence quantification, basin stability, bifurcation diagram, noise-induced chaos

## Abstract

This paper examines the nonlinear behavior of the generalized stochastic intermediate dispersive velocity (SIdV) equation, which has been widely analyzed in a non-noise deterministic framework but has yet to be studied in any depth in the presence of varying forcing strength and noise types, in particular how it switches between periodic, quasi-periodic, and chaotic regimes. A stochastic wave transformation reduces the equation to simpler ordinary differential equations to make soliton overlap analysis feasible to analyze soliton robustness under deterministic and stochastic conditions. Lyapunov exponents, power spectra, recurrence quantification, correlation dimension, entropy measures, return maps, and basin stability are then used to measure the effect of white, Brownian, and colored noise on attractor formation, system stability, and spectral correlations. Order–chaos transitions as well as noise-induced complexity are more effectively described by bifurcation diagrams and by Lyapunov spectra. The results of this experiment improve the theoretical knowledge of stochastic nonlinear waves and offer information that will be useful in the fields of control engineering, energy harvesting, optical communications, and signal processing applications.

## 1. Introduction

Nonlinear dynamical systems and soliton theory have become important foundations in both applied mathematics and theoretical physics. Phenomena such as chaos, pattern formation, self-organization, and localized waves frequently arise in these systems, and many of these features originate in mathematical models governed by nonlinear equations. Nonlinear dynamics provides a more accurate framework than linear methods for understanding complex behaviors, including climate fluctuations, population dynamics, and energy transfer in optical fibers and plasmas [1,2,3].

Recent studies on continuous soliton equations provide important insights, which are taken into consideration to contextualize the present results within the broader developments in soliton dynamics [4,5,6,7,8]. Among the interesting nonlinear wave structures is the soliton, a wave packet that does not change shape as it moves forward. KdV, NLS, and sine-Gordon equations are among the integrable systems in which solitons are found. Since they were discovered in the 19th century, their remarkable durability and the degree to which they behave like particles have attracted much interest in studying them. For decades, the study of soliton dynamics has contributed to research in fiber communications, Bose–Einstein condensates, condensed matter, and cosmology.

Recently, scientists have focused on how soliton-bearing systems interact with two primary kinds of stochastic perturbations: additive noise and multiplicative noise, which, respectively, represent external environmental fluctuations and internal system-dependent randomness. The world around us is almost always filled with noise, and many important systems naturally vary from one moment to the next. Therefore, the behavior of such systems depends not simply on a vague balance between stochasticity and chaos, but rather on how random perturbations influence the underlying deterministic attractors and modify the system’s stability landscape. For example, ref. [9] looked at how solitons perform in GRIN multimode fibers when introduced to noise and showed the effects as changes in the paths of solitons and the shape of their energy. In addition, ref. [10] studied dispersive solitons described by the Schrödinger–Hirota model using stochastic calculus and found that randomness could result in important transition changes in the stability of these solitons. Such studies underline that using stochastic methods is necessary when building computer models for nonlinear wave propagation, the main topic of this research on the generalized SIdV (stochastic integrable dispersive velocity) model.

There have been important developments in the wider field known as chaotic dynamics. Key works such as [11] have introduced a well-structured method for examining nonlinear time changes, bifurcations, and strange attractors. Deterministic chaos refers to complex but fully deterministic behavior that arises from extreme sensitivity to initial conditions, where small variations in starting points lead to vastly different trajectories over time. It is typically characterized by irregular, aperiodic motion and fractal structures in the phase space. Refs. [12,13,14] developed new approaches based on the use of Lyapunov exponents, Poincaré sections, and the reconstruction of the phase space. For detecting chaotic behavior in environments with significant random noise and multiple interacting elements, these methods remain indispensable. Recent studies, such as [15,16], have shown that in soliton-bearing equations, stochastic perturbations can either enhance or suppress chaotic behavior, thereby providing deeper insight into how nonlinear systems respond to random disturbances.

Nonlinear science also focuses heavily on learning from perturbation theory and attractor dynamics. Many nonlinear systems have multiple attractor states, called multistability, in which the final state is strongly affected by where and how the experiment started and by small changes in parameters. Whether the disturbances are precise or random, they may cause the system to go through transitions and display new types of behavior. This can clearly happen in forced mechanical oscillators [17], quartic oscillators [18], and systems with coupled neurons. Basin stability has emerged as a useful measure for calculating the level of resistance of attractors to small disturbances, developed only recently. Among other works, refs. [19,20] show that the geometry inside basins influences both the stability and predictability of multistable systems.

Other main subjects in nonlinear systems, including synchronization and the formation of patterns, have been explored by taking multiple approaches. Most importantly, ref. [21] explored how coupling and noise change the ability of oscillators to synchronize, and these ideas have been used in neuroscience and research on the power grid. The phenomenon of having the system show chaotic movement during a transition stage and then become steady again has been described in lasers and fluid flows, mainly through the work of [22,23].

For detecting and interpreting different kinds of time patterns in unstable systems, recurrence quantification analysis (RQA) has become the leading method. As first described by [24] and later improved by [25], RQA lets us study time series by examining repeating patterns in the phase space and turning them into graphical and numerical data. Recurrence analysis, with both plots and networks, has been found to successfully detect transitions, hidden-word patterns, and predictable periods in chaotic and stochastic systems alike. It has been improved by using measures from entropy and by looking at fractal dimensions.

As for soliton theory, the classical work [26,27] gives a detailed description of integrable systems and the interaction of coherent waves. Recently, for [28,29,30] scholars in the field working with higher-dimensional systems and considering scenarios with a stochastic component, it turned out that such structures of solitons are still possible. Interesting proven versions are also associated with studying how modulation instability affects waves when they are very slightly disturbed. Solving this issue is of great interest both in fluid dynamics and in nonlinear optics.

Chaotic Attractor Analysis has been greatly assisted by recent developments in entropy and correlation dimension methods, where experts use various statistical measures (e.g., permutation counts and correlation dimensions) to distinguish among three types of dynamics (periodic, chaotic, and stochastic). These techniques, when combined with recurrence plots and Lyapunov spectra, provide an excellent means of understanding complex nonlinear systems, and because of their application in both engineering and science, they are useful in many areas of contemporary research [31,32].

It is critical to understand the nonlinear dynamic systems’ features (chaotic behavior, multiple stable states, and complex basin structures) to assess their predictability, transition between different states, and sensitivity to their initial conditions. The *basin attractor plot* is used to visualize and study the structure of basins of attraction in the phase space [33], while the *basin stability* is used as a measure of the robustness of a system under perturbation [34]. Thus, the combination of geometric visualizations of chaotic behavior with quantitative stability measures [35] provides a bridge between qualitative and quantitative studies of nonlinear systems.

The study of *probability in chaotic systems* has been an active area of research in nonlinear science for many years. In recent times, research has focused on the synthesis of *noise and chaos in dynamic systems* with transitory or intermittent chaotic behavior [36,37]. Noise can be used to drastically alter the structure of attractors; initiate bifurcations; and introduce order using methods such as coherence resonance and synchronization by noise.

### Research Gap, Novelty, and Contribution

While a number of studies have examined both nonlinear dispersive equations and their stochastic counterparts [13,14,16,17], a number of these studies have been limited to studying either deterministic soliton dynamics or purely stochastic effects of noise. The collective impact of the higher-order nonlinear terms, the multiplicative noise, and the externally applied periodic driving on the interaction between solitons and the attractors they produce has not been well studied. Moreover, prior studies rarely provide a systematic phase-space analysis that connects stochastic perturbations, order–chaos transitions, and noise-induced complexity. These limitations highlight the research gap that this work aims to address.

This study is innovative in that it reduces the generalized stochastic SIdV Equation (1) to nonlinear oscillator models that take deterministic, forced, and noise-driven effects into account. Unlike other approaches, this work considers soliton overlap, stochastic regularization, and chaotic transitions simultaneously using a variety of analytical and numerical diagnostics.

The specific contributions of this article are summarized as follows:

- Reduced-order nonlinear oscillators with periodic forcing and stochastic perturbations are used to illustrate the generalized stochastic SIdV equation.
- Broad analysis of soliton overlap, multistability, attractor fluctuations, and order–chaos transitions in both deterministic and stochastic contexts is conducted.
- Chaotic features and stability structures are characterized by fractal measurements, power spectra, return maps, surrogate data, and recurrence analysis.
- Dynamical regimes are categorized using bifurcation diagrams alongside Lyapunov spectrum analysis, with an emphasis on noise-induced transitions.
- The results may find use in nonlinear wave control, energy transport in complex media, and noise-assisted signal processing.

The nonlinear behavior of the extended stochastic SIdV equation under different forcing amplitudes and noise types is still poorly understood, despite the fact that it is helpful for modeling intermediate dispersive velocity phenomena. Specifically, the mechanisms underlying the transitions from periodic to quasi-periodic and chaotic regimes, and the effects of white, Brownian, or colored noise on stability and attractor creation, are not well understood. This gap must be filled in order to advance the study of nonlinear dynamics, which has applications in control engineering, energy harvesting, signal processing, and optical communication.

Our results have applications in a number of physical and engineering fields. Considering the effects of stochastic perturbations on soliton stability and attractor behavior allows control engineers to design effective controllers for nonlinear fluctuating systems that allow for noise-tolerant functioning and targeted suppression of undesirable chaotic responses. By improving data integrity and minimizing signal distortion, an understanding of noise-induced transitions and consistency preservation under different stochastic regimes can aid in optimizing signal propagation via fiber optics for optical communication. Determining the characteristics of multistable states and noise-assisted transitions lays the groundwork for applying stochastic resonance in energy harvesting applications, increasing the efficiency of energy capture in systems susceptible to outside disturbances. These examples link theoretical modeling with practical implementation by demonstrating how the quantitative understanding of stochastic dynamics provided by the generalized SIdV framework can be directly applied to real-world nonlinear systems.

In this study, we study the development of unpredictable attractors, foundational probability mechanisms, and soliton stability depending on noise in order to study the stochastic fluctuations of a generalized SIdV equation. With the help of Lyapunov exponents, recurrence quantification, basin stability, and stochastic perturbations, our method offers a thorough explanation of noise-driven complexity.

The purpose of this work is to present a systematic analysis of the modified stochastic SIdV equation’s dynamical behavior. The mathematical analysis of the governing model is presented in Section 2, where the generalized stochastic SIdV equation is introduced in Section 2.1 and the corresponding dynamical system is derived in Section 2.2. The results and discussion are presented in Section 3 and are presented from a number of complementary angles. In particular, soliton overlap in deterministic and stochastic settings is studied in Section 3.1, and in Section 3.1.1 the uncertainty quantification and noise-induced soliton stability are analyzed. Meanwhile attractor dynamics and the order–chaos transition are the subject of Section 3.2. In Section 3.2 the attractor geometry and structural consistency are displayed. Section 3.3 discusses chaotic features and basin stability, while Section 3.4 analyzes power spectra and Section 3.4 compares noise effects quantitatively. Section 3.5 addresses validation of surrogate data and return maps, while Section 3.6 explores fractal structures and chaotic dynamics. Section 3.7 employs recurrence plots for assessing dynamical regimes. In Section 3.7 the autocorrelation and memory decay analysis under stochastic forcing is studied. Meanwhile, Section 3.8 emphasizes complexity, stability, and chaos brought on by noise. The proposed perturbed Hamiltonian system is examined in Section 3.11, while bifurcation diagrams and Lyapunov spectrum analysis are displayed in Section 3.9. Section 3.9 is added to analyze the numerical consistency of the stochastic quartic oscillator. Section 3.10 is related to the variance-based sensitivity analysis of the stochastic SIdV equation. The comparative context with previous studies is discussed in Section 5. A summary of this study’s conclusions and recommendations for additional research are provided in Section 5.

## 2. Mathematical Analysis

### 2.1. Governing Model

In this work, we analyze the generalized stochastic SIdV equation [14]:

(1) $$v_{s}+\left(2\left(1-\gamma \right)v+\left(1+\gamma \right)\frac{v_{\eta \eta }}{v}\right)v_{\eta }-\mu v_{\eta \eta \eta }=\xi v\frac{dW(s)}{ds},$$

where v(η,s) is a real function regarding space η and time *s*, γ and μ are constants, and ξ scales the stochastic perturbation. Here, W(0)=0 and W(s) are typical Wiener processes with independent Gaussian increments. For ξ=0 and γ=μ=−2, Equation (1) reduces to the classical KdV equation [13,14].

We adopt a traveling-wave ansatz capturing both deterministic and stochastic effects [16,17]:

(2) $$v\left(\eta ,s\right)=P\left(\eta \right)\;e^{\xi W\left(s\right)-\frac{\xi^{2}}{2}s},\;\eta =\beta y-\omega s,$$

wherein the wave profile is represented by P(η). The exponential factor ensures that E[v(η,s)] evolves deterministically by eliminating random drift in expectations.

The random wave transform has a key physical and analytical role in separating the regular wave structure from the noise fluctuations. Physically, this transform acts as a multiplicative stochastic modulation, eliminating the mean drift arising from random perturbations and allowing a study of how noise affects the amplitude and phase of coherent structures, without distorting the overall wave envelope. It enables the mapping of a stochastic partial differential equation to a deterministic one with modified coefficients, allowing a detailed phase-space, stability, and bifurcation analysis to be undertaken. In addition to the present study, this transformation has a very large field of application in the various types of stochastic and nonlinear systems where the consideration of coherence and randomness is of great importance, such as soliton propagation in optical fibres under the influence of thermal fluctuations, wave–current interaction in ocean dynamics, and energy transport processes in plasma and magneto-hydrodynamic systems. Thus, the transformation provides both the theoretical basis and computational convenience for separating out and analyzing the deterministic backbone of the complex stochastic wave phenomena.

Substituting (2) into (1) and averaging over noise yields the reduced deterministic ODE:

(3) $$-\beta^{3}\mu P^{\prime \prime }-\omega P^{\prime }+\beta P^{\prime }\frac{\beta^{2}\left(\gamma +1\right)P^{\prime \prime }}{P}+2\left(1-\gamma \right)P=0.$$

Integrating once after multiplying by *P* gives

(4) $$\begin{array}{cc} PP^{\prime \prime }-d_{1}{\left(P^{\prime }\right)}^{2}+d_{2}P^{2}+d_{3}P^{3}+d_{4} & =0,\\ \mathrm{where}\;\left\{\begin{array}{cc} d_{1} & =\frac{1+\mu +\gamma }{2\mu },\\ d_{2} & =\frac{\omega }{2\beta^{3}\mu },\\ d_{3} & =\frac{\gamma -1}{\beta^{2}\mu },\\ d_{4} & =-\frac{c_{0}}{2\beta^{3}\mu }. \end{array}\right. \end{array}$$

For phase-space analysis, we define $Z=P^{\prime }$:

(5) $$\left\{\begin{array}{c} P^{\prime }=Z,\\ Z^{\prime }=d_{1}\frac{Z^{2}}{P}-d_{2}P-d_{3}P^{2}-\frac{d_{4}}{P}. \end{array}\right.$$

Using the first integral method, a conserved “energy-like” quantity is

(6) $$Z^{2}=MP^{2d_{1}}-\frac{2d_{3}}{3-2d_{1}}P^{3}+\frac{d_{2}}{d_{1}-1}P^{2}+\frac{d_{4}}{d_{1}}.$$

### 2.2. Dynamical System

For $d_{1}=5/2$, Equation (6) reduces to a quintic nonlinear oscillator:

(7) $$\left\{\begin{array}{c} P^{\prime }=Z,\\ Z^{\prime }=\frac{5M}{2}P^{4}+\frac{3d_{3}}{2}P^{2}+\frac{2d_{2}}{3}P. \end{array}\right.$$

Including a periodic forcing term gives the perturbed system:

(8) $$\left\{\begin{array}{c} P^{\prime }=Z,\\ Z^{\prime }=m_{4}P^{4}+m_{2}P^{2}+m_{1}P+f_{1}\cos\left(M(\eta )\right), \end{array}\right.$$

where $m_{4}=\frac{5L}{2},\;m_{2}=\frac{3d_{3}}{2},\;m_{1}=\frac{2d_{2}}{3}$, and M(η)=qη is the phase of the drive.

This can be extended to a 3-dimensional system including the phase dynamics:

(9) $$\left\{\begin{array}{c} P^{\prime }=Z,\\ Z^{\prime }=m_{4}P^{4}+m_{2}P^{2}+m_{1}P+f_{1}\cos\left(M\right),\\ M^{\prime }=q, \end{array}\right.$$

Finally, the perturbed noisy dynamical system is

(10) $$\left\{\begin{array}{c} P^{\prime }=Z,\\ Z^{\prime }=m_{4}P^{4}+m_{2}P^{2}+m_{1}P+f_{1}\cos\left(M(\eta )\right)+\sigma \;\xi \left(\eta \right), \end{array}\right.$$

where σ scales the stochastic input ξ(η).

In this case, *P* represents the wave’s amplitude, *Z* its derivative, with *M* the periodic drive’s phase. Through the exponential component of (2), the multiplicative stochastic component introduces *stochastic regularization*, stabilizing expectation while possibly causing *noise-driven transitions* as well as stochastic resonances based on σ, ξ, and the system parameters (γ,μ).

## 3. Results and Discussions

### 3.1. Soliton Overlap in Deterministic and Stochastic Settings

Comparison of soliton structures under deterministic and stochastic conditions gives useful information on the nonlinear wave retention or degradation of coherence in the presence of random variations. Conventional analyses tend to seek to obtain soliton solutions of the analytical type or to individually investigate how they evolve with time. Nevertheless, such methods are not commonly applied to measure the energy or structural dissimilarities of different soliton families. To fill this gap a visual and quantitative structure for the examination of the extent of congruence among various soliton solutions is given: *soliton overlap*. Pairwise squared amplitude overlaps can be compared to evaluate the sensitivity of the intrinsic profiles of various solitons to stochastic perturbations, e.g., Gaussian, singular, and implicit complex. This technique offers a fresh diagnostic view going beyond analytical derivations, which allows the determination of soliton structures, which are stable and resistant to noise, and provides a rich diagnostic of the processes of coherence retention and transition in stochastic nonlinear wave systems.

This paper compares three analytical solutions to the stochastic (noisy) and deterministic versions of the main equation, i.e., Equation (1) from [38], which are also analytical solutions of this equation. Their structural homogeneity and noise resistance are investigated with the help of pairwise squared amplitude maps and three-dimensional visualizations of overlap. Specific attention is paid to the degree of overlap between solitons, which also provides a direct understanding of common dynamics and their convenient exchangeability in nonlinear modeling, including Gaussian, peakon, and singular solitons associated with the stochastic SIdV equation. While these analytical derivations are extensively documented in the literature, our study introduces a distinctive comparative methodology by superimposing these contemporary soliton solutions to evaluate their stability in the face of stochastic influences and their structural similarity. This overlap-based approach gives a measurable way to compare the coherence of soliton families and lets you directly compare deterministic along with noisy regimes, which is more than just the usual solution derivation. Our overlapped soliton approach offers an enhanced physical understanding of noise resilience through inter-soliton correlation within stochastic nonlinear systems, as demonstrated by [38]. The produced overlap patterns exhibit more complex dynamical correspondence than those previously characterized.

The three distinct solutions considered are as follows.

**Gaussian Soliton (Sol1):** The Gaussian soliton solution has the functional form

$$v\left(y,s\right)=A\exp\left[-\frac{\beta }{8\omega^{3}}{\left(\zeta -\zeta_{0}\right)}^{2}\right]\Delta ,\;\Delta =e^{\xi W\left(s\right)-\frac{\xi^{2}}{2}s},\;\zeta =\beta y-\omega s,$$

where *A*, β, ω, $\zeta_{0}$, and ξ are the defining parameters (A=1.2, β=2, ω=1, $\zeta_{0}=0$, and ξ=0 in the deterministic case). The limited, stable wave packet represented by this nonsingular solution decays exponentially with ζ→∞. It is computationally cheap to evaluate due to its analytical form, and evaluation in noisy conditions is made possible by the stochastic factor Δ.

**Singular Soliton (Sol2):** The expression for the singular soliton is

$$v\left(y,s\right)={\left(\frac{\sqrt{3/2}}{\sqrt{C}\left(\zeta -\zeta_{0}\right)}\right)}^{2/3}\Delta ,$$

with parameters *C*, ω, β, $\zeta_{0}$, and ξ (C=1, ω=1, β=−1, $\zeta_{0}=0$, and ξ=0 for the deterministic case). This solution exhibits a singularity at $\zeta =\zeta_{0}$, reflecting a self-similar blow-up, and decays as a power law $∼{\left(\zeta -\zeta_{0}\right)}^{-2/3}$ for large ζ. It is computationally beneficial for both deterministic as well as stochastic situations since it is analytically tractable.

**Implicit Complex Soliton (Sol3):** The implicit complex soliton with the amplitude V(ζ) is determined implicitly via a nonlinear relation:

v(y,s)=V(ζ)Δ,

depending on parameters *a*, *b*, *c*, ω, β, $\zeta_{0}$, and ξ (a=2, b=0, c=1, ω=1, β=−1, $\zeta_{0}=0$, and ξ=0 in the deterministic case). The implicit form of V(ζ) determines the asymptotic properties of this solution, which may show singular performance if V(ζ) approaches a critical value (e.g., V(ζ)→c). It can be understood as a phase-transition-like characteristic with a complex nonlinear structure, and unlike each of the other two solitons, it necessitates numerical root-finding.

**Figure 1:**
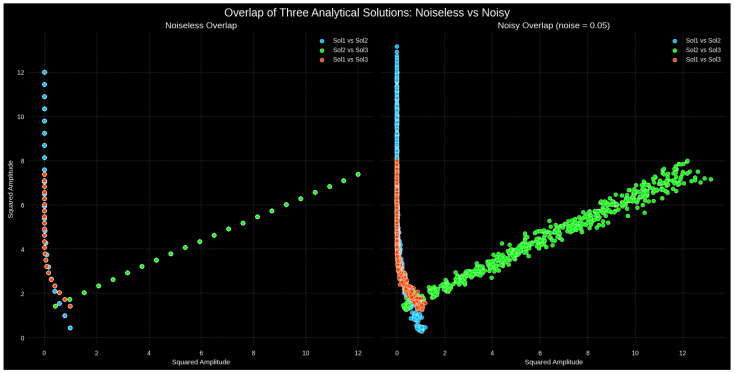
The three soliton solutions’ pairwise squared amplitude overlaps. (**Left**): Noiseless case. (**Right**): Noisy case with σ=0.05.

The pairwise squared spectrum overlaps regarding the three soliton solutions—the Gaussian, singular, and implicit complex solutions—are displayed in Figure 1. These overlaps show sensitivity regarding stochastic disturbances and structural similarities. In the noiseless case, the soliton overlaps between Gaussian and singular and Gaussian and implicit complex exhibit rigid clustering at low amplitudes, indicating significant agreement regarding their low-energy environments, but divergence during higher amplitudes, indicating deviations from their nonlinear growth development. Conversely, the singular vs. implicit complex solitons exhibit a roughly linear relationship throughout the entire amplitude range, suggesting that these two solutions share a consistent structural resemblance and comparable dynamical properties. The entire patterns of the overlap become more and more dispersed with random perturbations, as well as when we add the value of σ=0.05. However, the singular–implicit pair demonstrates its resistance to noises in that it shows the same linear trend. The increased variance of the overlaps of the Gaussian soliton implies that its characteristics are more vulnerable to random perturbations.

Two soliton patterns $\psi_{i}\left(x\right)$ and $\psi_{j}\left(x\right)$ have a squared amplitude overlap that may be expressed as

$$O_{ij}=\int |\psi_{i}{\left(x\right)|}^{2}\;{\left|\psi_{j}\left(x\right)\right|}^{2}\;dx,$$

It gauges how closely the energy densities of the two solitons coincide. The scatter diagrams in Figure 1, which display this overlap pointwise across the spatial domain, offer a quantitative illustration of the theoretical overlap measure. Rather than reproducing analytical soliton solutions that were previously identified in the literature, this approach offers a comparison framework to assess the way the present soliton families interact and preserve coherence via stochastic forcing. By photographing the degree of structural similarity and energy correlation among different soliton forms, this overlap-based model offers a new diagnostic perspective for studying resilience, coherence loss, and stability transitions in stochastic nonlinear wave systems.

**Figure 2:**
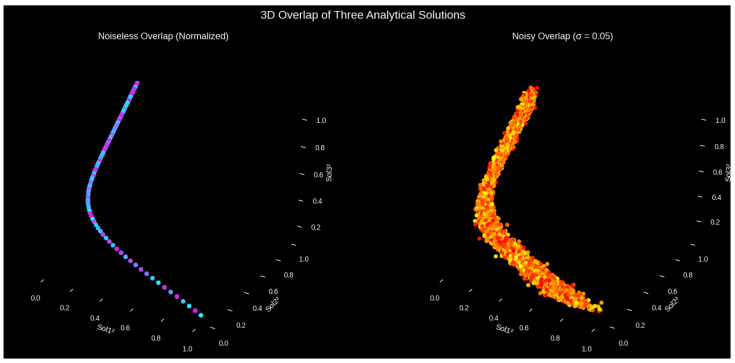
Three-dimensional squared amplitude overlap among Sol1, Sol2, and Sol3. (**Left**): Noiseless case (normalized). (**Right**): Noisy case with σ=0.05.

Figure 2 presents the 3D squared amplitude overlap of the noiseless and noisy cases of the three soliton solutions, namely the Gaussian soliton, singular soliton, and implicit complex soliton. When we are in the noiseless state, it is clear that the points belong to a one-dimensional range in the three-dimensional space, and this means that all the solutions are connected by a fundamental functional structure and a strong mutual relationship. Although there is variation in the amplitude properties of the individual solutions, such alignment indicates that the individual solutions are closely related in their aggregate dynamics and that there is inherent consistency in the nonlinear behavior of the system. At lower stochastic noise levels, with a noise value of σ=0.05, the manifold is less compressed and its effects are visible, but the overall tube-like formation can be observed. This dispersion does not affect the implicit correlations of the three soliton solutions, as shown by the fact that the 3D structure is still present, which proves that the relationships and functional dependencies of these solutions are robust against noise. This analysis provides some insight into the way in which noise influences the higher-dimensional interconnections between complex soliton solutions by indicating both the stability of the solution set under ideal conditions and the sensitivity of the solution set under stochastic forcing.

The simulations were run with the help of such scientific computing libraries as **NumPy** to perform numerical calculations, **sympy** of the library **SciPy** to perform basic mathematics and solve equations, and **Matplotlib** and **Seaborn** to perform visualization using **Python 3.x**. The computational parameters were the spatial and time intervals of ξ∈[−6,6] along with t∈[−1,1], respectively, discretized with 50 and 25 steps, respectively. In order to test robustness, additive Gaussian noise with the strength 0.05 was employed.

The nonlinear ordinary differential equations are incorporated with the fourth-order Runge–Kutta method, which is applied to solve_ivp in the SciPy library so as to ensure uniformity of the numerical experiments. During the simulations, a fixed step size was used; the value of the step size was Δξ=0.01, and it was observed that this value did not violate the Courant–Friedrichs–Lewy (CFL) stability criterion. The stability parameters as well as the integration framework were kept similar across all sections to facilitate comparability. Convergence testing was further performed by dividing the difference Δξ in the square of the error by half, and the results showed that the amplitude and the trajectories of the phase showed negligible changes and confirmed the accuracy and reliability of the numerical system.

The singular and implicit complex solitons are coherent and stable to noise, but the three solitons have much geometric overlap and common dynamics. This justifies the use of solitons of limited size and nonlinear wave resilience to noise in practical applications and emphasizes the significance of overlap as an important measure of nonlinear wave coherence [33,39,40].

#### 3.1.1. Uncertainty Quantification and Noise-Induced Soliton Stability Analysis

We seek a rigorous understanding of the effects of the random fluctuations and parameter uncertainties on the stability and long-term behavior of nonlinear systems that exhibit soliton solutions. The coherence of the waves, the persistence of amplitude, the onset of complex dynamics, and so on may all be significantly altered in actual physical situations by fluctuations in system properties and external noise. To capture these effects, stochastic and uncertainty-based analyses have to be applied alongside deterministic modeling. To present a complete framework of soliton consistency and sensitivity to parameter changes and the predictability of model outcomes under random effects, this section includes both a multiplicative noise analysis and a quantification of uncertainty (UQ) (comprising Monte Carlo simulations and Polynomial Chaos Expansion (PCE)) [41,42].

**Figure 3:**
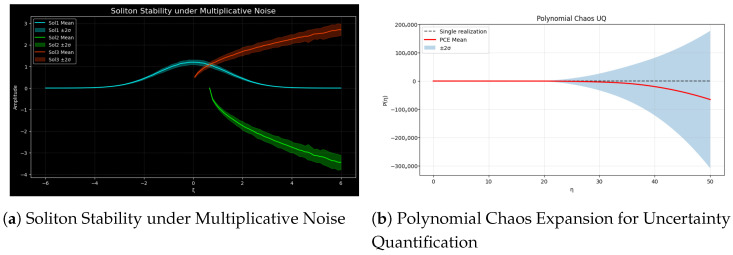
The impacts of noise and parameter variability on system dynamics are highlighted by soliton stability under stochastic perturbations (**a**) and parametric uncertainty propagation via PCE (**b**).

As shown in Figure 3, the analysis of the model will be used to show two analyses of the system robustness and uncertainty propagation. Although panel (b) applies the Polynomial Chaos Expansion (PCE) to measure the effect of the uncertainty in the parameters, panel (a) looks at the statistical stability of the three analytically independent soliton solutions in the presence of multiplicative noise. Collectively, these studies provide a comprehensive numerical study of uncertainty-based complexity, soliton robustness, and stochastic sensitivity in nonlinear mechanisms.

(a) Soliton Stability Under Multiplicative Noise

Fundamentally, the stability of solitons is an essential problem in understanding the mechanics of disordered systems. Essentially, the stability of solitons is a critical issue with regard to the dynamics of disordered systems. Panel (a) shows the mean and ranges of three soliton solutions under multiplicative stochastic perturbations in intensity, defined by the mean of the solutions, (Sol1) Gaussian soliton, (Sol2) singular soliton, and (Sol3) implicit complex soliton, under multiplicative stochastic perturbations in intensity ε=0.05. Each soliton was perturbed as

$$v_{\mathrm{noisy}}\left(\zeta \right)=v\left(\zeta \right)\;\left[1+\varepsilon \;\eta \left(\zeta \right)\right],$$

where η(ζ) and ε=0.05 represents random fluctuations. A total of 100 Monte Carlo realizations were used to compute the mean and the mean value ±2σ to test the stability. Sol1 is the most stable because of its smooth nonsingular profile, Sol2 is highly sensitive and divergent around the value of the parameter $\zeta =\zeta_{0}$, and Sol3 experiences mild phase drift and deformation. As a consequence, soliton durability is extremely reliant on its geometric form, which illustrates the relationship between stochasticity, variation, and nonlinearity. This is numerical and can be used to derive explicit noise-dependent solitons as far as the modified Kudryashov method or Riccati expansion is concerned [42].

(b) Polynomial Chaos Expansion for Uncertainty Quantification

In panel (b) intrinsic fluctuations are represented by four different Gaussian variables: ($m_{4},m_{2},m_{1},f_{1}$). A PCE surrogate model fitted on 50 Monte Carlo samples predicts the mean trajectory (red) and the band of ±2σ (blue), unlike a deterministic example (dashed). The growing P(η) confidence interval shows the nonlinear sensitivity of the system and how any uncertainties in parameters will exponentially grow the output variance, even in the case of a very small uncertainty in the parameters of the system [41]. The stochastic integration is stabilized numerically and is parallelized to ensure the stability of the stochastic integration. Finally, it can be concluded that stochastic forcing and parameter uncertainty collaborate to maintain soliton reliability, as demonstrated in Figure 3a,b above. Monte Carlo and PCE analyses of different resilience patterns in Sol1-Sol3 are found to give a consistent framework in which the stability, sensitivity, and predictive confidence in nonlinear situations are evaluated.

### 3.2. Attractor Dynamics and Order–Chaos Transition

**Figure 4:**
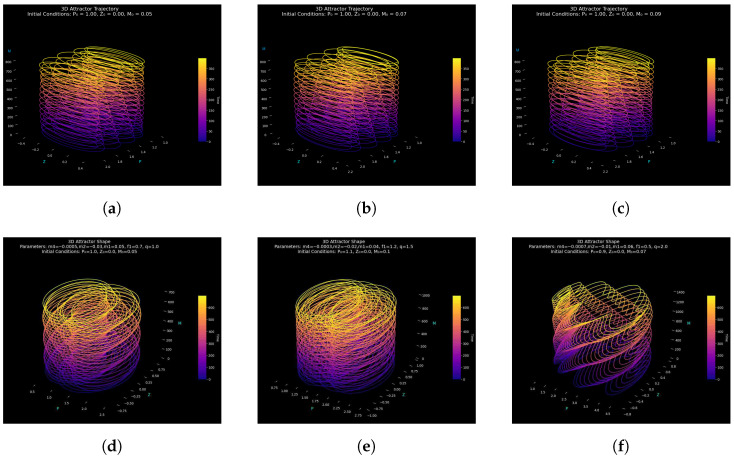
Three-dimensional attractor structures under quasi-periodic forcing. Two-dimensional phase projections and time series of the perturbed system (9).

**Figure 5:**
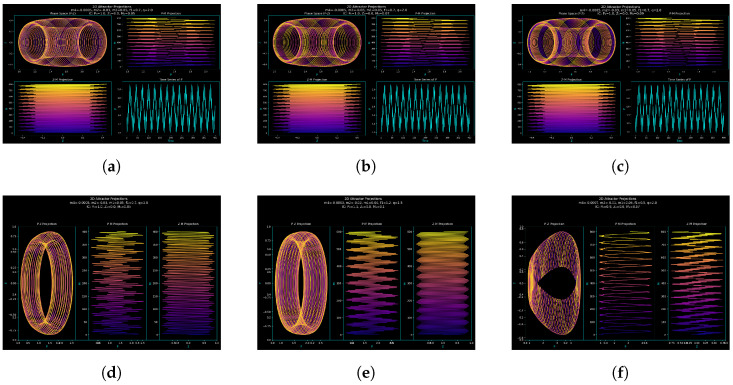
Two-dimensional phase projections and time series of the perturbed system (9).

The complexity of the quasi-periodically generated system (9) is assessed in this study. We look at the structure of its attractors in different scenarios: the three-dimensional attractor structures under quasi-periodic forcing and two-dimensional phase projections and time series of the perturbed system, i.e., showing sensitivity to forcing phase and amplitude as well as shifting from quasi-periodicity to chaos [21]. These findings are not only theoretically intriguing but also practically applicable because they enable the development of systems that can either promote or prevent chaos, depending on the desired outcome. For instance, modifying the chaotic settings can ensure secure signal masking in communication systems or increase the efficiency of energy transfer in nonlinear harvesters, both of which rely on predictable but modifiable chaotic responses. These dynamics are shown in Figure 4 and Figure 5, with interpretations explained below.

Figure 4 uses three-dimensional attractors to show the geometry of the system’s long-term dynamics. Trajectory layering and direction are slightly changed by changing the initial phase $M_{0}$ (Figure 4a–c), exhibiting sensitivity to initial conditions, a characteristic of nonlinear systems. The attractor geometry is more drastically altered by changes in parameters like $m_{4}$, $m_{2}$, $m_{1}$, $f_{1}$, and *q* (Figure 4d–f), which go from predictable ring-like patterns (stable quasi-periodicity) to entangled orbits (incipient chaos) and, finally, to deformed, asymmetric trajectories that are suggestive of fully developed chaos. These findings demonstrate that while parameters drive global transitions from order to chaos, initial conditions control fine attractor structure.

Python and the relevant scientific libraries, including Matplotlib for visualization, NumPy for numerical computations, and SciPy’s odeint solver for integrating the system of differential equations, were used to conduct the simulations. To guarantee numerical stability and precise resolution of the attractor dynamics, key computational parameters were selected, including the integration range and time step (Δt=0.01).

The same flexible step control along with tolerance were used in all parameter regimes to ensure consistency with other numerical studies. For stiff and weakly chaotic regimes, odeint depends on the Runge–Kutta 4(5) algorithm, which guarantees stability under the Courant–Friedrichs–Lewy (CFL) condition. Transient dynamics were eliminated to eliminate starting bias, and each simulation was iterated for $10^{5}$ time steps to guarantee convergence to the steady-state attractor. The results’ numerical robustness and reproducibility were ensured by validation tests that used tighter temporal discretization to verify that the attractor geometry and Lyapunov exponents, alongside phase-space topology, stayed constant.

Figure 5 displays time series and two-dimensional phase projections of the perturbed system (9). The 3D attractors’ quasi-periodic properties are shown in Figure 5a–f. Closed loops and sinusoidal signals are included in Figure 4a–c and Figure 5a–c. The dynamics, however, progressively shift as the parameters are altered (Figure 5d–f), exhibiting regular elliptical loops (quasi-periodic), denser structures with modulated time series (onset of complexity), and, at the end, irregular signals and distorted projections that demonstrate fully developed chaos. The findings demonstrate that initial conditions and external forcing have an impact on attractor geometry and temporal evolution.

At low forcing amplitudes and frequencies, the system displays smooth quasi-periodic attractors; at higher forcing, however, increasingly complex structures emerge, which ultimately degenerate into chaos via a quasi-periodic path governed by $f_{1}\cos\left(M\right)$. This transition is confirmed by comparisons between 2D projections and 3D trajectories (Figure 4 and Figure 5). Sinusoidal loops pass through modulated forms before becoming chaotic irregular patterns. While parameters $f_{1}$ and *q* govern global transitions from order to chaos, the beginning phase $M_{0}$ has a significant impact on fine attractor layering. In addition to revealing fundamental mechanisms of order–chaos transitions, the study of attractor morphologies provides a functional basis for engineering systems where chaotic modulation or suppression can be applied systematically, allowing a direct connection between the observed behavior and feasible control strategies.

#### Attractor Geometry and Structural Consistency

**Figure 6:**
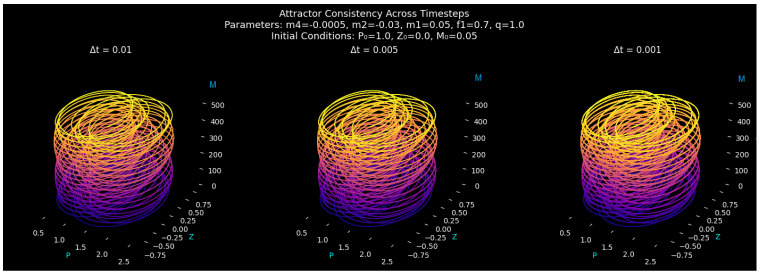
Attractor consistency across numerical time steps for system (9). The three reconstructions (Δt=0.01,0.005,0.001) exhibit nearly identical helical structures, confirming geometric and topological stability under time step refinement.

Three integration steps, Δt=0.01,0.005,and0.001, were used to model the attractor evolution while maintaining the same initial conditions and parameters in order to confirm the geometric fidelity of system (9) under numerical discretization. Figure 6 displays the comparative attractor reconstructions, emphasizing how the system’s spatial organization remains consistent across different temporal resolutions. The attractors in the (P,Z,M) phase space of the system, which represent finer time steps, have nearly the same global envelope, retaining the helical stratification and cylindrical folding of the forced quartic oscillator. There is no dependence of the trajectory curvature or periodic stacking on time step reduction, which means that the geometry of the attractive set is not dependent on the selection of the time step Δt. This invariance ensures that no arbitrary geometric deformations are caused by numerical discretization and demonstrates that the nonlinear interaction between the periodic drive and the quartic stiffness is resolved exactly even with coarser time steps.

In all the three integration steps, the orbits appear as spirals along the *M*-axis, devoid of fake loops or fragmentations. In the topology of the underlying manifold dynamics of slow modulation, rapid oscillation can be seen to be statistically stable, as the distance between the loops is essentially constant and the density of the attractor is completely uniform. The granularity of time steps, but not topological deformation, is only revealed by the slight changes in the color gradient, which mark the time advancement. Consequently, the manifold coherence of the attractor of the quartic oscillator is preserved in the enhancement of integration. When further analyzing Figure 6 regarding attractor time step consistency, there is no flattening, fragmentation of the trajectories, or false oscillations that could be indicative of integration artifacts. Even though it is somewhat rougher, the attractor at the smaller time steps Δt=0.01 still has the same phase-space envelope as the better solutions at smaller time steps. The trajectory’s smooth color consistency provides additional evidence of the minimal discretization flaws. As a result, rather than numerical noise or instability, the visualization faithfully captures the inherent dynamics of the system (9).

System (9) defines a deterministic quartic oscillator that exhibits good structural and numerical consistency. Across discretization scales, the attractor morphology, manifold integrity, and visual continuity do not vary, indicating that the observed geometric dynamics are system-wide and not the result of the integration method.

### 3.3. Chaotic Features and Basin Stability

The basin attractor maps illustrate how initial conditions shape the long-term behavior of the nonlinear system (9), with parameters $m_{1}=-1.5$, $m_{2}=2.5$, $m_{3}=0.8$, $f_{1}=0.7$, and q=1.618 (golden ratio). Multistability and sensitivity to initial conditions is emphasized between discrete and continuous representations, which show the existence of periodic and chaotic attractors, fractal basin boundaries, and quasi-periodic regions. Basin stability, which quantifies attractor robustness, measures the fraction of beginning conditions that approach each attractor, bypassing the significance of local stability. The quantification of resilience during perturbation, attempts to project dominating attractors, and the recognition of chaotic attractors by the fractal basin are techniques that provide information on the dynamics in physics, engineering, and biology. A non-polynomial forcing element of the dynamics, such as the element of cos(M), is added that enhances the dynamics by offering the theoretical and visual evaluation of the stability landscape and the game of chaos and resilience.

**Figure 7:**
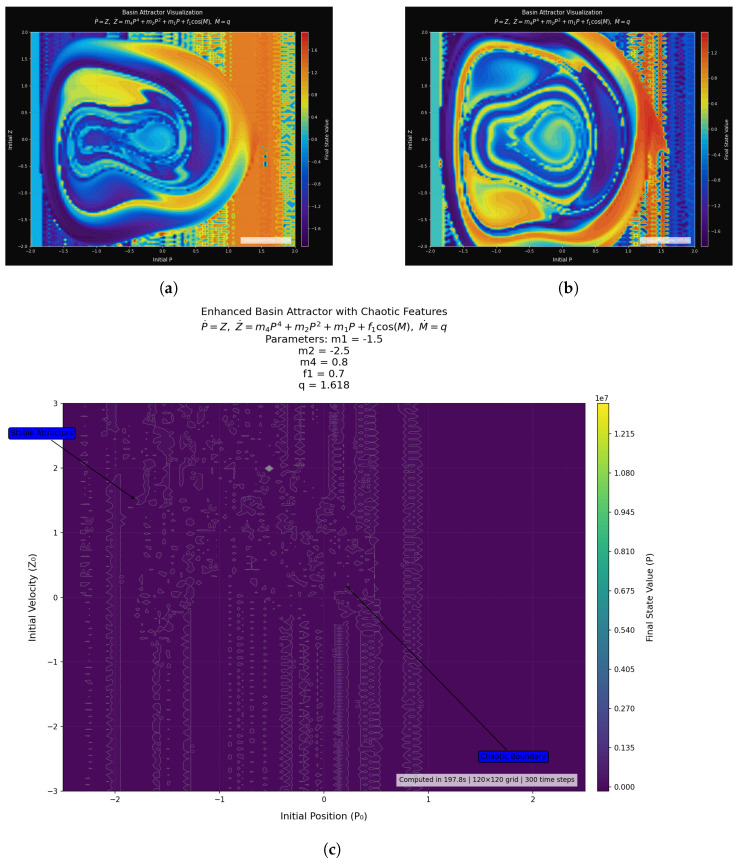
Basin attractor plots showing final states via color gradients and annotated regions distinguishing chaotic and stable domains. (**a**) Binary classification of final states (**b**) Color gradient indicating final state value (**c**) Color-coded map with annotated chaotic and stable regions.

Figure 7a shows the discrete basin attractor map, where the areas in black and white indicate the various end states. This binary, threshold-based mapping is a grouping of the initial circumstances that converge at the attractor. The fact that the system is extremely sensitive to initial conditions is illustrated through the fragmentary and interlaced structures, where even slight variations can lead to deviation of paths to other attractors. The irregular structures of the map that indicate the presence of multiple coexisting attractors profoundly interwoven with the basin boundaries highlight the complexity and unpredictability of the system.

Continuous basin mapping (Figure 7a) is a method of mapping that displays the final value of P or other state-related values using a color gradient. Unlike nested rings and smooth gradients, which display quasi-periodic areas with deterministic, predictable curves, sudden color changes represent chaotic areas that are very sensitive to initial conditions. This illustration is an excellent visualization of the chaos and regularity mechanisms of the phase space and manages to isolate the regular and erratic dynamics.

A final state annotated basin map of initial condition values on the plane of (P, Z) is presented in Figure 7b. The color bar gives the final values of *P*. Whereas the chaotic borders produce noisy effects with a lot of variability, showing vulnerability to the initial conditions, stable attractors appear in the form of uniform coloring, serving as a continuous convergence. The simulation draws attention to the combination of stability and unpredictability between starting configurations by obtaining a satisfactory resolution to represent the transitions between order and chaos of the system in a grid of size 120 120 and time steps 300.

The system is remarkably multistable, as it can be seen that multiple attractors exist or the final states heavily rely on the initial conditions of the system. The boundaries between fractal basins display chaos, yet forcing at the golden ratio of q = 1/phi creates a quasi-periodicity, with non-chaotic yet complicated dynamics. Complex borders are represented by chaotic saddles that indicate temporary chaos as pathways are attracted to stable attractors. Basin attractor plots give a picture of the phase space in the world at large scale; the stable areas are smooth, and the disorganised areas are fractal or tangled basins. Although subplot Figure 7c emphasizes spirals associated with resonance and chaotic saddles, the bottom heat map compares stable attractors with chaos boundaries. The several overlapping attractors and interconnecting basins are illustrated in subplots 1 and 2 in Figure 7a,b. The process rediscovers multistability, bifurcations, and attractor dominance by exploring a broad space of initial conditions, providing both probabilistic and geometric knowledge about world dynamics. Although it is computationally intensive and sensitive to sample density, it gives a sound framework of determining the robust and weak attractors and guiding control tactics.

#### Basin Stability Analysis

**Figure 8:**
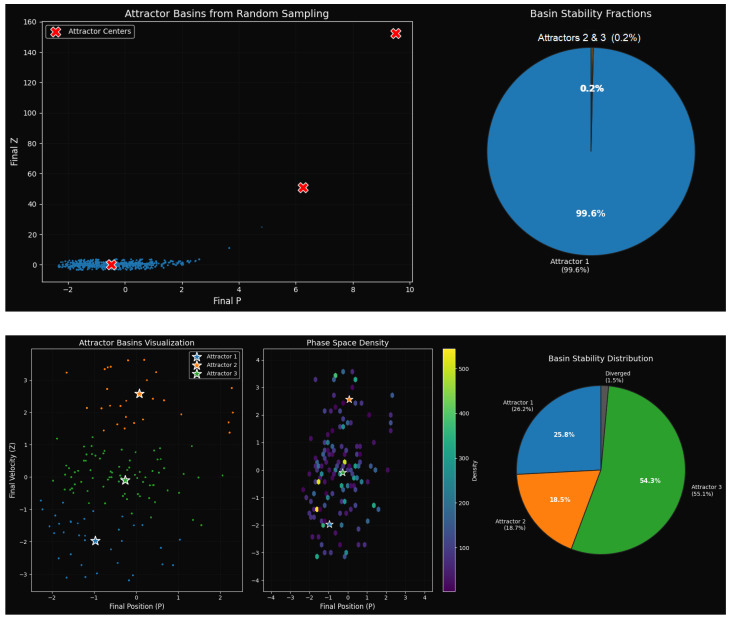
Basin stability visualizations: (**Top**)—dominance of a single attractor; (**Bottom**)—multistability with phase space density and attractor distribution.

Basin stability is a probabilistic attractor robustness metric that relates the initial circumstance volume to global dynamics. Complex systems have multistability, fractal basins with boundaries, and the dominance of attractors. Figure 8 shows two basin stability tests with different sampling densities of system (9), each having distinct attractor behavioral patterns. When combined they can give both qualitative and quantitative data on the resistance and multistability of basins.

Figure 8’s top panel illustrates how one attractor dominates the multistability landscape. Three attractors live together, but they are very unevenly stable. Attractor 1 at (P,Z)=(−0.442,0.081) dominates with a radius of attraction of ≈0.999 and basin stability of 99.73%, indicating global stability. Conversely, Attractor 2 at (9.500,152.338) and Attractor 3 at (6.248,50.944) exhibit incredibly narrow basins with negligible stabilities of 0.07% and 0.20% with radii of ≈0.027 and ≈0.045. The scatter plot (top left) displays trajectories clustering around Attractor 1, while the pie chart (top right) validates its near-total dominance. Therefore, the system effectively behaves as monostable despite formal multistability, requiring strong perturbations or finely tuned initial conditions to transition to minor attractors. Theoretically and practically, Attractor 1 is a robust default state that shows how multistable systems can reduce to a single operational attractor.

The bottom panel of Figure 8 illustrates a balanced multistability scenario. From the analysis of 14,779 valid samples (with 1.47% diverged trajectories), the system is shown to host three competing attractors. Compared to strongly dominant cases, this configuration exhibits a more balanced distribution, with Attractor 3 at (P,Z)=(−0.261,−0.091) emerging as the most probable state (55.06% basin stability, relative attraction =1.000). In contrast, Attractor 1 (−0.968,−1.970) and Attractor 2 (0.078,2.575) capture 26.21% and 18.73% of initial conditions, respectively. Their effect is smaller and yet significant, as shown in the comparative values of attraction (0.476 and 0.340 of Attractors 1 and 2). The divergence rate of 1.47 percent is a demonstration of the importance of stability restrictions in applications to the real world because it highlights the effects of boundaries when the trajectories leave the finite phase space.

Multistability is confirmed by visual diagnostics of Figure 8. The fractal basin boundaries (bottom left) indicate sensitive partitions, phase space density (bottom center) indicates clustering about attractors, and the pie chart (bottom right) indicates the basin occupancies and divergence. They jointly display perturbation-susceptible metastable states, are compatible with both controlled modes, and are critical to noise-induced transitions and hysteresis. This two-mode approach offers a predictive foundation for the evaluation of multistability, resilience, and control means of probabilistic stability analysis and geometric basin representation.

### 3.4. Analysis of Power Spectra

Power spectrum analysis enhances time-domain methods by revealing the frequency-dependent energy distribution and identifying periodic, quasi-periodic, and chaotic states and effects in time series analysis [26,43]. It discovers chaos or randomness, determines the most important frequencies, and depicts the process of decay with respect to the attractor structures. The power spectral density (PSD) of a signal, x(t), is provided as

(11) $$P\left(\omega \right)=\lim_{T\to \infty }\frac{1}{T}{\left|\int_{-T/2}^{T/2}x\left(t\right)e^{-i\omega t}dt\right|}^{2}.$$

**Figure 9:**
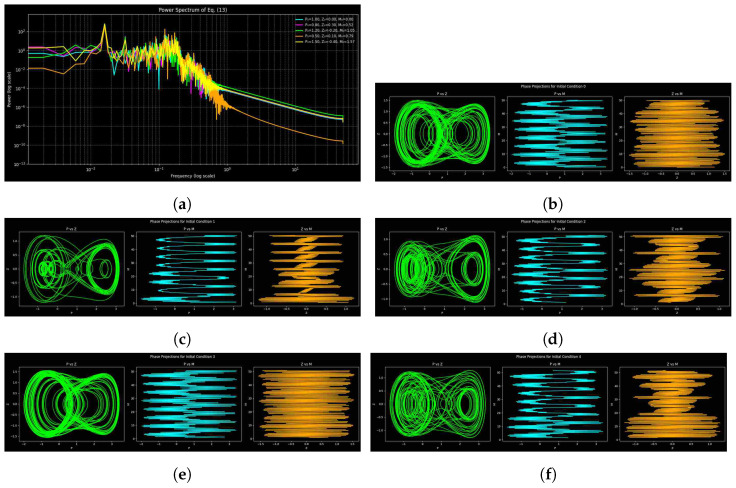
(**a**–**f**) Phase space and power spectra for five initial conditions (parameter set 1, Δη=0.01, [0,500]); peaks indicate quasi-periodic components within chaos.

**Figure 10:**
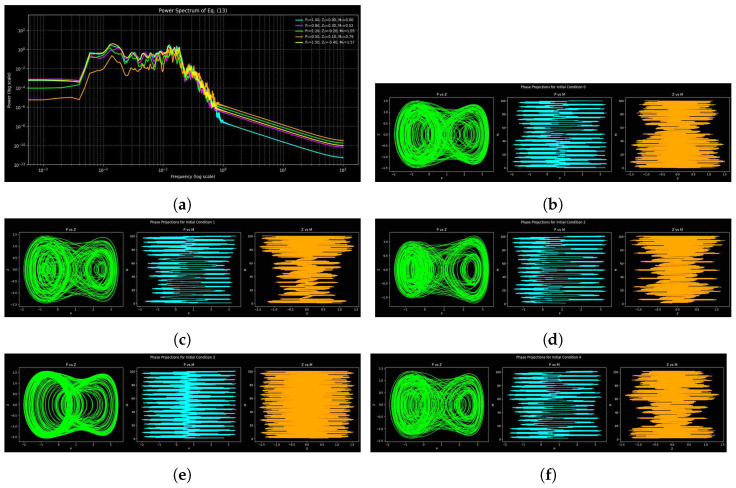
(**a**–**f**) Phase space and power spectra for five initial conditions (parameter set 2, Δη=0.005, [0,1000]); broader peaks and denser spectra indicate stronger chaotic behavior.

**Figure 11:**
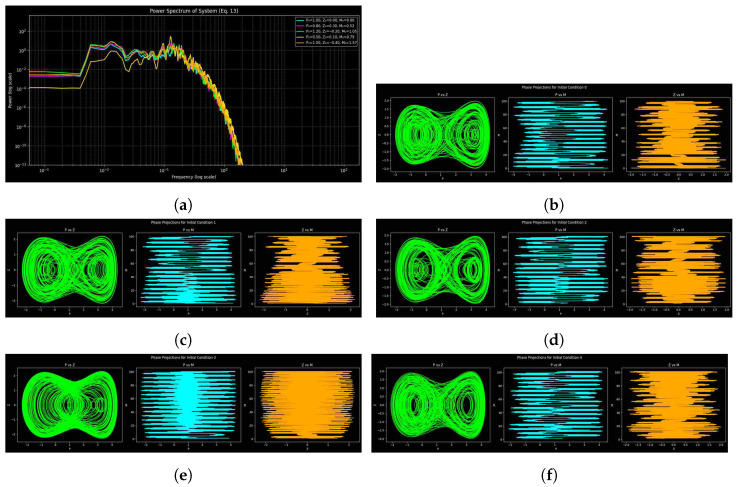
(**a**–**f**) Power spectra along with phase space with five initial conditions (modified parameter set 3, difference of Δη=0.005, [0,1000]); narrower peaks correspond to reduced chaos and, perhaps, to quasi-periodicity.

By comparing phase portraits and power spectra for various parameter sets of system (9), one can gain a better understanding of dynamical transitions by highlighting spectral peaks, decay, and spread. Figure 9, Figure 10 and Figure 11 show phase-space trajectories and corresponding spectra of system (9) under three parameter sets, each tested with five initial conditions: (1.0,0.0,0.0), (0.8,0.3,π/6), (1.2,−0.2,π/3), (0.5,0.1,π/4), and (1.5,−0.4,π/2). They include an array of phase shifts, amplitudes, and velocities. Broadband spectra are chaotic or unpredictable, sharp peaks are periodic and expanded ones are quasi-periodic, and the pattern of decay is dissipative. Table 1 contains parameter sets, integration periods, and time periods to ensure reproducibility and to highlight the importance of numerical resolution. But, there is sensitivity to parameter change, as Figure 11 reveals, and parameters are shared in Figure 9 and Figure 10 but with different step sizes and lengths; successful deterministic chaos is observed with filamentary trajectories in the phase space.

The dynamics are shown in Figure 9 with parameter set 1. The power spectrum has a noisy profile with tiny peaks that points to mild chaos or quasi-periodicity, whereas low-frequency fluctuation highlights sensitivity to initial conditions. The repetitive spectral peaks indicate that chaos is periodic by nature, with few frequencies prevailing in the organized dynamics. The attractor geometry is resolved enough with the selected step of integration (0.01).

Figure 10 shows the dynamics with parameter set 2. Increasing the length of the simulation and resolution provides smooth spectra with huge low-frequency peaks representing dominating oscillatory modes, but the high-frequency contents decay gently, following an approximate power-law tendency. A smaller range of spectral curves implies an increase in near-attractor dynamics. There is higher complexity of the trajectory in the phase space compared to set 1, which implies that it is more chaotic. Wider and less distinct spectral peaks indicate a greater overlap of the modes and a greater complexity in the entropy, which indicates that the longer the simulation and the finer the discretization, the more sensitive to initial conditions the simulation becomes and the more irregular chaotic structures appear.

For parameter set 3, the dynamics are shown in Figure 11. The spectrum of power demonstrates a weak low-frequency response and exponential high-frequency decay, which implies more strong nonlinear interactions and energy stability. Whereas overlapping spectral curves suggest convergence to a shared attractor, narrow, discrete peaks suggest less chaotic mixing and partial quasi-periodicity. Phase-space trajectories confirm improved stability, showing that weakly chaotic or quasi-periodic behavior with more distinct spectral organization is favored by modifications in parameters (*a*, *b*, and *c*).

Spectral analysis reveals that as the time of simulation and the choice of parameters changes, the weakly chaotic and noisy dynamics (Figure 9) change to smooth and predictable ones (Figure 11). Whereas set 3 includes less chaotic elements, which means that it is more likely to enter quasi-periodic activity, set 2 (Figure 10) magnifies chaos and nonlinear interactions with smaller time steps. The spectral and phase-space analysis are complementary, as seen by their comparison; spectral analysis gives frequency content, and phase-space plots show the geometry of the trajectories. They offer a detailed understanding of the dynamical regimes and sensitivity of the parameters together.

#### Quantitative Comparison of Noise Effects

**Figure 12:**
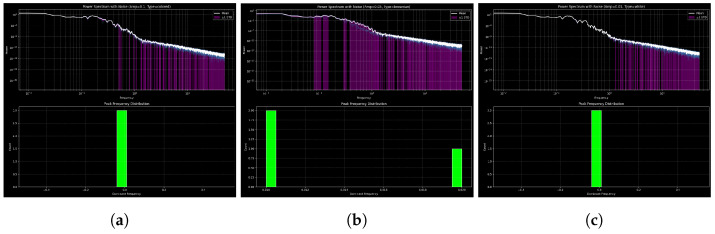
Power spectrum and peak frequency distribution under different noise types. (**a**) White noise (Amp = 0.1) (**b**) Brownian noise (Amp = 0.05) (**c**) Colored noise (Amp = 0.05).

In this work, the the relationship between different types of noise and the dynamics of the system under consideration (10) are explored [30]. These are the findings of white, Brownian, and colored noise presented in the panels in Figure 12a–c. The bottom of each of the panels displays a histogram of the most common (peak) frequencies in all realizations and shows the effect of the type of noise on spectral properties and frequency distributions. The uppermost plot of each panel shows the power spectrum of the ensemble in log–log form with the mean of the ensemble (white) and a standard deviation of ±1 (purple shading).

The response of the 3D nonlinear oscillator to various stochastic forcings across multiple realizations is shown in Figure 12. The PSD is displayed on a log–log scale in the top panel of each subplot, and the dominant frequency histogram is shown in the bottom panel. This allows for frequency-domain characterization and stability evaluation, which is interpreted by peak-frequency dispersion, spectral sharpness, and the persistence of dominant oscillations.

Strong, uniform oscillations are indicated by the PSD’s smooth decay and narrow variability under white noise (amplitude 0.1, Figure 12a) and the histogram’s sharp, centered peak [27]. In addition to adding strong inter-realization variability and enhancing low-frequency power, Brownian noise (amplitude 0.05, Figure 12b) creates a bimodal histogram with peaks close to zero and finite frequencies, which indicates destabilization and irregularity. A broader central histogram peak, a somewhat preserved oscillatory coherence, and a moderately elevated low-frequency PSD are the characteristics of colored noise (amplitude 0.05, Figure 12c).

In general, the comparison shows that white noise maintains strong oscillations, Brownian noise makes things unstable and bimodal, and colored noise gives rise to intermediate, partially coherent dynamics.

A summary of how white, Brownian, and colored noise affect amplitude, spectra, oscillations, and stability can be found in Table 2. Colored noise produces intermediate behavior, Brownian noise causes drift and instability, and white noise maintains coherence.

Statistical, spectrum, and phase-space analyses show how nonlinear oscillators react to noise and changes in parameters: colored noise maintains intermediate coherence, Brownian noise destabilizes through low-frequency drift, and white noise preserves oscillations. Spectrum decay and peak frequencies, which are measures of robustness, indicate that the nature of noise is very important to stochastic stability. This plays an important role in simulating and controlling oscillators in a random environment.

The numerical simulations were conducted using **Python 3.x** with key scientific computing libraries, including **NumPy** for numerical operations, **SciPy** for numerical integration using the **solve_ivp** function with the RK45 method, and *Matplotlib* for visualization. Solver tolerances were set to *rtol = $1\times 10^{-5}$* and *atol = $1\times 10^{-7}$* to ensure numerical accuracy in the integration of the dynamical system.

In order to preserve numerical stability in both deterministic and stochastic regimes, the time step and integrating span were also carefully selected. Gaussian white noise with variance $\sigma^{2}=0.05$ was created for stochastic runs using numpy.random.normal, and statistical reliability was ensured by ensemble averaging over 50 realizations. In the Itô sense, the Euler–Maruyama scheme made sure that the noise was properly integrated and convergence was established by way of time series, phase portraits, and Lyapunov exponents between step sizes.

White noise preserves the primary phases of the nonlinear oscillator as well as permits regular energy exchange across system components since its uncorrelated, zero-mean oscillations equally affect all frequencies, maintaining oscillatory coherence from a mechanical standpoint. Whereas colored noise preserves a certain level of coherence between the white noise oscillations and the Brownian drift, the presence of Brownian noise in biform spectra and instability is due to the addition of low-frequency drifts by Brownian noise. The oscillator resonance dictates its special spectral and histogram characteristics as it acts to amplify or suppress noise frequencies depending on resonance. These effects can be mathematically explained in accordance with the system’s response function.

### 3.5. Return Maps and Surrogate Data Validation

Return maps offer a distinct structure for monitoring the evolution of nonlinear dynamics from order to chaos and stochasticity [19,44]. By plotting x(t+τ) against x(t), they show the geometry of the attractor, the memory of the system, and how predictable it is. Simulations over t∈[0,100] (10,001 points) and delays τ∈1,10,50,100,500,1000,6000,9000,9500,9900,10,000 captured correlations across scales for the attractor system (9) with parameters $m_{4}=-1.0$, $m_{2}=0.0$, $m_{1}=10.0$, $f_{1}=1.0$, and q=1.5 and initial state [1.0,0.0,0.0]. Moderate forcing encourages regime transitions, the linear drive maintains oscillations, and the nonlinear term stabilizes large amplitudes. The resulting return maps differentiate between dynamical states: chaos creates folded fractal sets [20], quasi-periodicity displays secondary structures, stochastic dynamics scatter into uncorrelated clouds, and periodic motion produces discrete points or loops. As the system transitions from equilibrium to randomness, these patterns show how memory and predictability deteriorate.

**Figure 13:**
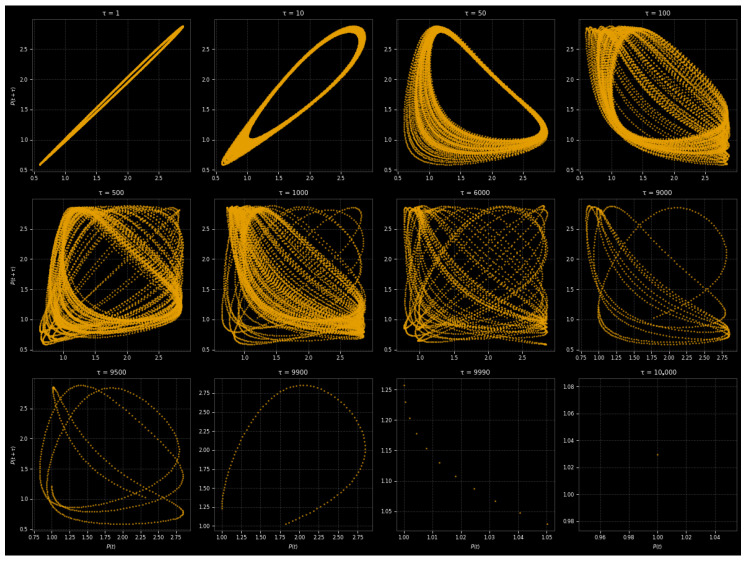
Return maps of the attractor system (9) for different delays τ, showing the change from periodic order to chaotic complexity and then to random, memoryless behavior.

The system gradually loses order as the delay τ increases, as shown by the return maps in Figure 13. Short delays (τ=1–100) cause the dynamics to change from a distinct linear correlation at τ=1 to smooth periodic loops at τ=10, folded structures at τ=50 that indicate nonlinear distortions, and finally fractal-like geometries at τ=100, which are indicative of chaos. The attractor broadens further with intermediate delays (τ=500–1000). At τ=500, several folds appear, and by τ=1000, the system exhibits dense, complex structures characteristic of fully developed chaos. Predictability breaks down and memory effects decrease at long delays (τ=6000–10,000). The structure starts to break down by τ=6000; trajectories split into thin bands at τ=9000–9500; and the maps take on the appearance of random scatter at τ=9900–10,000, indicating almost complete decorrelation.

Return maps depict chaos and decreasing predictability over time, as deterministic systems lack consistency with increasing delay.

With a time step of **0.01**, a total time of **100**, and tolerances rtol = $1\times 10^{-6}$ and atol = $1\times 10^{-6}$, simulations were carried out in **Python 3.x** employing **SciPy**’s solve_ivp for integration, alongside **NumPy** and **Matplotlib** for calculation and visualization.

The system’s susceptibility to stochastic changes and initial circumstances was assessed through ensemble simulations. The stochastic differential equations were incorporated in the *Itô sense* employing the Euler–Maruyama method, guaranteeing correct handling of noise contributions. Gaussian white noise was produced using numpy.random.normal alongside specified variance. Temporal stability and accuracy were ensured by comparing trajectories across smaller time increments (Δt=0.005) for each integration run. Poincaré sections along with Lyapunov spectra were calculated for systems with chaotic as well quasi-periodic behavior in order to verify the stability of numerical results across integration schemes and evaluate the attractor properties. To produce statistically sound results and describe the impact of stochastic disturbances on system dynamics, ensemble averaging over several realizations was carried out.

**Figure 14:**
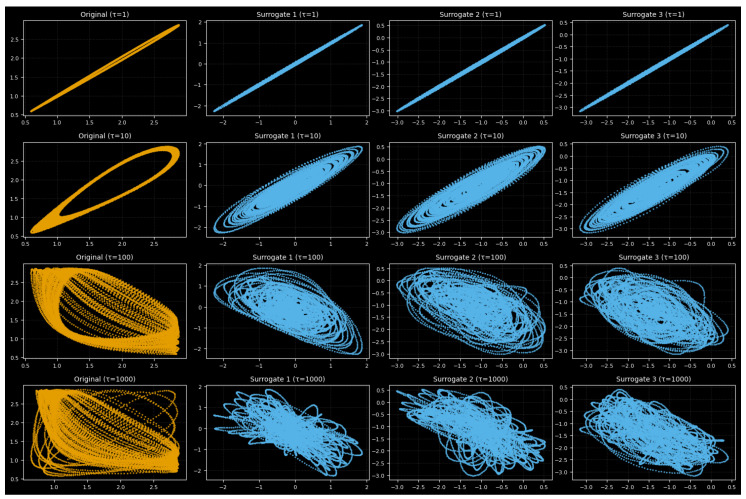
The deterministic nature of system (9) is revealed by return maps for delays τ=1,10,100,1000: structured originals vs. randomized surrogates.

The original system and phase-randomized surrogate data were compared in order to evaluate determinism (Figure 14). Both seem to be almost linear for τ=1, indicating short-term correlations. The original displays periodic loops at τ=10, whereas surrogates produce smoother ellipses. While surrogates deteriorate into diffuse clouds, chaotic folding appears in the original at τ=100. The original maintains weak clustering by τ=1000, but the surrogates lose structure as they become fully randomized.

These discrepancies are supported by key dynamical metrics in Table 3: surrogates maintain their lower correlation value with minor variations, while the original system exhibits a higher correlation dimension and recurrence rate and longer decorrelation time. This proves that deterministic chaos, not noise, is the source of the complexity.

Surrogate analysis confirms that these patterns originate from deterministic nonlinear processes rather than random fluctuations, and the return maps and metrics collectively show a progression from predictable to chaotic and finally stochastic dynamics with increasing τ.

### 3.6. Chaotic Dynamics and Fractal Structure

To investigate the long-term behavior of the forced quartic oscillator, we employ a combined qualitative–quantitative approach. While fractal-dimension analysis quantifies the attractor’s complexity and separates chaotic from regular regimes, phase-space visualization shows the attractor’s geometry [44]. It is shown that the system (8) evolves in a chaotic regime when studied with the following parameters: $m_{4}=-0.015$, $m_{2}=0.75$, $m_{1}=-0.25$, $f_{1}=1.2$, and Q=1.8. We characterize the underlying strange attractor and diagnose the irregular dynamics using fractal-dimension estimation and phase-space reconstruction.

**Figure 15:**
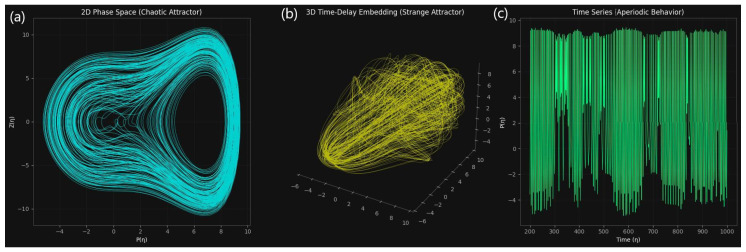
Phase-space and temporal dynamics of system (8): (**a**) 2D phase portrait, (**b**) 3D time-delay embedding, and (**c**) aperiodic time series P(η).

Figure 15 illustrates the qualitative features of the system. The two-dimensional phase portrait (P,Z) revealed a densely folded trajectory with intricate layering, lacking closed orbits or toroidal structures. This indicated that the long-term dynamics are not governed by periodic or quasi-periodic attractors but instead by a strange attractor. The reconstructed attractor in the three-dimensional time-delay embedding exhibited the stretching and folding mechanism typical of chaos, suggesting the presence of fractal geometry in the underlying phase space. Complementarily, the time series P(η) showed strongly irregular oscillations with intermittent amplitude modulation and no recurring patterns, reinforcing the absence of long-term periodicity.

**Figure 16:**
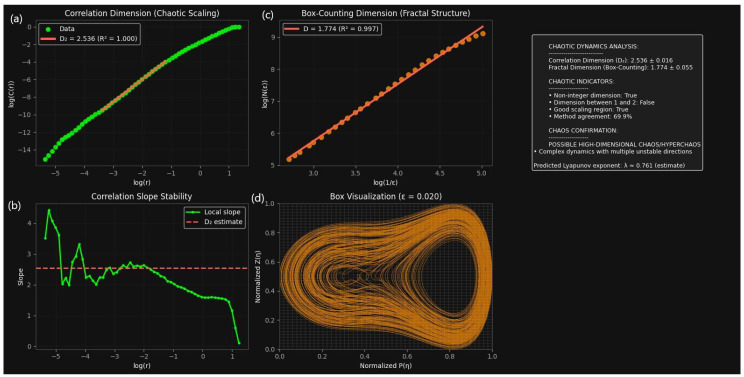
Fractal-dimension analysis of system (8): (**a**) correlation dimension, (**b**) local slope, (**c**) box-counting dimension, and (**d**) grid visualization.

To substantiate these observations, Figure 15 presents fractal-dimension estimates derived from correlation and box-counting methods. The correlation dimension was determined as $D_{2}=2.536\pm 0.016$, extracted from a well-defined scaling region with $R^{2}\approx 1.0$. The local slope analysis confirmed this estimate by exhibiting a stable plateau, validating the robustness of the fractal scaling. Independently, the box-counting dimension yielded $D_{\mathrm{box}}=1.774\pm 0.015$ with $R^{2}=0.997$, in good agreement with the correlation dimension despite minor methodological differences as shown in Figure 16. Both measures converged on non-integer values, ruling out regular attractors and confirming the fractal nature of the dynamics. The value of $D_{2}$ between 2 and 3 further suggested that the attractor resides on a moderately low-dimensional manifold, implying that at least three effective degrees of freedom are required to capture the system’s evolution.

Taken together, the results provide clear evidence that system (8) evolves in a chaotic regime. The irregular time series, strange attractor geometry in the phase space, and consistent non-integer fractal dimensions ($D_{2}\approx 2.54$, $D_{\mathrm{box}}\approx 1.77$) collectively establish deterministic chaos as the governing mechanism. Beyond confirming chaos, the analysis quantifies the attractor’s intrinsic dimensionality, showing that the dynamics unfold on a moderately low-dimensional manifold. These insights are crucial for classifying the oscillator’s long-term behavior, informing reduced-order modeling strategies, and situating this system within the broader class of nonlinear forced oscillators where predictability is fundamentally limited despite deterministic governing equations.

The simulations were conducted using **Python 3.x** with key libraries, including **SciPy** for numerical solvers (specifically the solve_ivp function with the **DOP853** method) and **sklearn** for regression and data preprocessing. The system was integrated with a time step of **0.02** and a total simulation time of **1000 s**, utilizing tight tolerances (rtol = $1\times 10^{-10}$ and atol = $1\times 10^{-10}$) for accurate results. **NumPy** was used for numerical operations and **Matplotlib** for visualization.

Furthermore, to ensure numerical consistency, convergence tests were performed by varying both the integration step size and solver tolerances. The DOP853 scheme’s adaptive step control was validated through comparison with RK45 for selected trajectories, confirming identical attractor geometries and invariant measures. For stochastic simulations, Gaussian white noise was generated using numpy.random.normal, and the stochastic differential equations were integrated in the *Itô sense* using the Euler–Maruyama method. Ensemble averaging over multiple realizations was performed to ensure statistical reliability of the results. For extended simulations, data-driven diagnostics such as correlation dimension and permutation entropy were computed, enabling quantitative verification of the system’s dynamic stability and chaotic regimes.

### 3.7. Dynamical Regimes via Recurrence Plots

**Figure 17:**
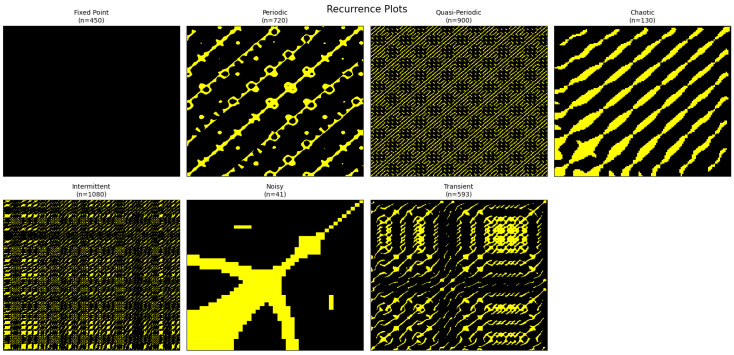
Recurrence plots of the perturbed noisy system (10), illustrating equilibrium, periodic, quasi-periodic, chaotic, intermittent, noisy, and transient regimes.

Recurrence plots provide a strong framework for the analysis of nonlinear and stochastic dynamical systems by highlighting the recurrence of states in the phase space, thus uncovering hidden patterns that remain obscured in unprocessed time series [31,32,45]. Their function transcends mere visualization; they facilitate the identification of patterns, correlations, and transitions across various dynamical regimes, rendering them essential for the characterization of both short- as well as long-term activities [36,37]. In Figure 17, recurrence plots of the perturbed noisy system (10) display various dynamical regimes, including equilibrium (fixed-point convergence), periodic oscillations, quasi-periodic motion on tori, chaotic evolution with fractal structure, intermittent chaos-order switching, noise-dominated fluctuations, and transient states evolving toward long-term attractors.

Figure 17’s geometric signatures offer a methodical way to categorize dynamics: homogeneous dark blocks that represent convergence to equilibrium are fixed points; long, regularly spaced diagonals that emphasize precise state repetition are produced by periodic regimes; chaotic regimes show fragmented, irregular diagonals that indicate sensitive dependence on initial conditions; and quasi-periodic regimes show diagonals with subtle modulations, which are characteristic of toroidal dynamics. In addition, more complex behaviors can be identified: transient dynamics reveal evolving patterns where early irregular structures gradually organize into stable recurrences as the system approaches an attractor; noisy or stochastic systems produce scattered point-like structures where correlations are suppressed by randomness; and intermittent dynamics appear as alternating ordered diagonal segments with irregular patches, reflecting transitions between laminar and bursty phases. Recurrence plots, when combined, provide quantitative measurements of stability, complexity, and dynamical change as well as qualitative insights into the entire range of dynamical behaviors, including equilibrium, oscillatory, chaotic, intermittent, noisy, and transitional.

The simulations were conducted using **Python 3.x** with the **SciPy** library for numerical integration, specifically using the solve_ivp function with the **LSODA** method for stiff problems. **NumPy** was used for numerical operations and **Matplotlib** for plotting, including an optimized recurrence plot computation using StandardScaler for data normalization. Key computational settings include a time step of **0.02** and a total simulation time of **1000 s**, with **tolerances** set at rtol = $1\times 10^{-6}$ and atol = $1\times 10^{-6}$ for accuracy.

Additionally, the LSODA solver automatically switched between stiff and non-stiff regimes, ensuring stability across varying noise intensities and nonlinearities. Each simulation was cross-validated against the RK45 scheme to confirm consistency in phase-space trajectories and attractor reconstruction. For stochastic simulations, Gaussian white noise was generated using numpy.random.normal, and the stochastic differential equations were integrated in the *Itô sense* using the Euler–Maruyama method. Ensemble averaging over multiple realizations was performed to ensure statistical reliability. Recurrence quantification measures were computed using a fixed threshold distance based on normalized Euclidean metrics to maintain comparability across datasets. This hybrid numerical–statistical framework enhances both the reliability and reproducibility of the reported results.

#### Autocorrelation and Memory Decay Analysis Under Stochastic Forcing

The autocorrelation function (ACF) provides a quantitative measure of memory retention in stochastic dynamical systems. For the generalized stochastic SIdV oscillator, the ACF was computed from ensemble-averaged realizations of the main state variable over a range of noise strengths (σ=0.001–0.3). The corresponding correlation time $\tau_{c}$ was estimated as the integral of R(τ) up to its first zero crossing, representing the characteristic timescale over which the system retains information about its initial state.

**Figure 18:**
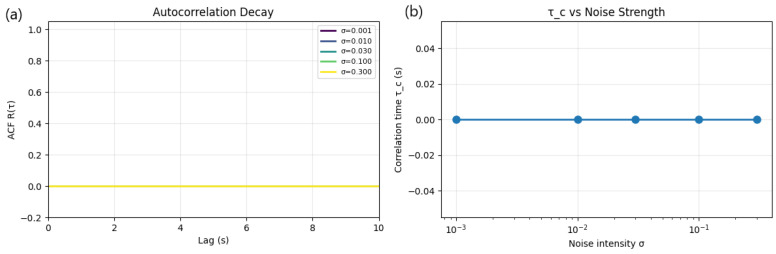
Autocorrelation decay in the stochastic SIdV system: (**a**) normalized functions R(τ) for varying noise σ and (**b**) correlation times $\tau_{c}$ versus σ, showing minimal memory retention.

Figure 18 shows two complementary diagnostics: (left subplot) the normalized autocorrelation decay curves R(τ) for different σ and (right subplot) the dependence of correlation time $\tau_{c}$ on noise strength. Across all tested cases, the autocorrelation rapidly decays to zero within a negligible lag, yielding $\tau_{c}\approx 0$. This indicates that the stochastic SIdV system exhibits almost instantaneous loss of temporal memory under all considered noise intensities.

Physically, this behavior suggests that the underlying nonlinear dynamics are strongly dominated by stochastic forcing rather than deterministic attractor memory. The absence of slow decay or long correlation tails implies that random perturbations disrupt the coherence of the system’s phase trajectory almost immediately, leading to uncorrelated fluctuations even at very weak noise levels. Consequently, the SIdV dynamics in this regime are effectively Markovian, with negligible temporal persistence.

This finding quantitatively confirms that, for the studied parameter range, stochastic forcing overwhelms intrinsic memory effects, resulting in a memoryless chaotic diffusive regime. Future work could extend this analysis near bifurcation thresholds or under lower forcing amplitudes, where non-trivial correlation times and stochastic resonance effects may emerge.

Simulations in **Python 3.10** using **NumPy**, **SciPy**, and **Matplotlib** employed the **Euler–Maruyama** scheme for SDEs, with a **0.02** time step, **100 s** total duration, and **eight Monte Carlo realizations** per noise level, using independent Gaussian noise seeds for statistical robustness.

Validated Euler–Maruyama integration with smaller time steps, i.e, (Δt=0.01), and ensemble averaging ensured numerical stability, convergence, and reliable, reproducible quantification of noise-induced transitions in system dynamics.

### 3.8. Noise-Induced Complexity: Stability and Chaos

We examine the chaotic behavior of a nonlinear quartic oscillator in deterministic and stochastic contexts in this study in terms of how noise influences the behavior of the system, i.e., phase-space analysis, Lyapunov exponents, and entropy. The noisy system is a perturbed noisy system , i.e., (10), which implies displacement P(η), velocity Z(η), stiffness coefficients $m_{4},m_{2},m_{1}$, forcing amplitude $f_{1}$, frequency *Q*, noise intensity shown by Sigma, i.e, σ, and Gaussian white noise ξ(η). Stability under stochasticity was quantified using ensemble-averaged variances and stochastic Lyapunov spectra across varying σ, distinguishing regimes of bounded fluctuations, divergence, and noise-sustained coherence.

**Figure 19:**
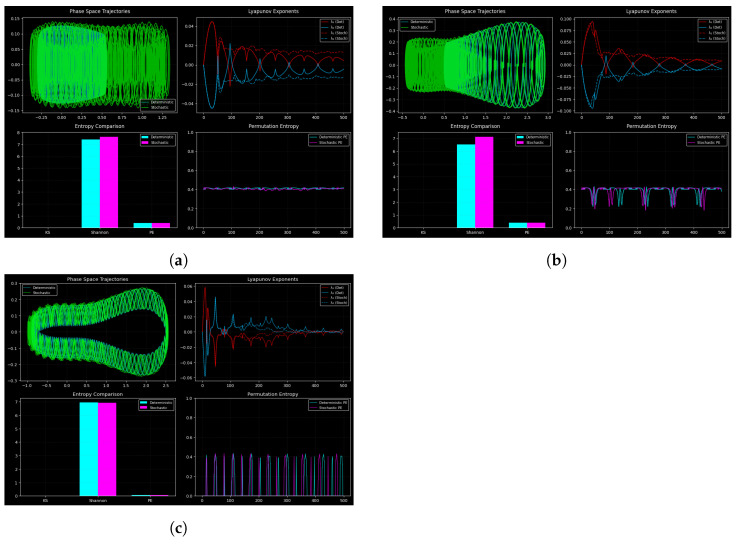
Deterministic vs. stochastic: phase-space trajectories, Lyapunov exponents, entropy comparison, and permutation entropy. (**a**) $m_{4}=-0.01$, $m_{2}=0.02$, $m_{1}=-0.01$, $f_{1}=0.3$, Q=π, and σ=0.1. Simulation parameters: η∈[0,500] with step size Δη=0.01, initial conditions [0.5,0.0], and ensemble size n=5. (**b**) $m_{4}=-0.003$, $m_{2}=0.02$, $m_{1}=-0.01$, $f_{1}=0.6$, Q=π, and σ=0.1. Simulation parameters: η∈[0,500] with step size Δη=0.01, initial conditions [0.5,0.0], and ensemble size n=5. (**c**) $m_{4}=-0.005$, $m_{2}=0.02$, $m_{1}=-0.003$, $f_{1}=0.2$, Q=π, and σ=0.05. Simulation parameters: η∈[0,500] with step size Δη=0.01, initial conditions [0.3,0.1], and ensemble size n=5.

By comparing deterministic and stochastic regimes using phase-space trajectories, Lyapunov exponents, and entropy-based metrics, we examine the perturbed system (10) in Figure 19. σ=0 denotes deterministic dynamics, while σ>0 denotes stochastic dynamics, where noise introduces extra complexity that is captured by permutation entropy.

Phase-space trajectories are displayed in the top-left panels of Figure 19, which contrast stochastic (green) and deterministic (cyan) dynamics. In toroidal quasi-periodic motion in nonlinear oscillators, deterministic trajectories are characterized by smooth closed loops [24] with intensity of noise Sigma. Noise causes stochastic trajectories to exhibit some slight deviation, yet they retain the underlying geometry, which is toroidal and dynamically robust. The system preserves its basic structure despite perturbations, as can be seen by the fact that quasi-periodicity remains in case of weak noise.

The two largest Lyapunov exponents (top-right panels of Figure 19) (denoted by the Greek letters ($\lambda_{1},\lambda_{2}$)) characterize the exponential separation of neighboring trajectories and are typically used as measures of chaos [46], with the top-right panel of Figure 19 ($\lambda_{1}$ in red and $\lambda_{2}$ in blue) showing slightly positive values in the deterministic case (solid curves), which is weak chaos or near quasi-periodicity, but otherwise the value is close to zero, which is transverse stability. Noise changes sensitivity to initial conditions, as the small perturbations in the values of the parameter under stochastic forcing (dashed lines) and reduced changes in the value of the dampened mode further evidence. The exponents are bounded over the range of the tested values of σ, implying that noise does not destabilize the system but, on the contrary, moves it towards the weak chaos that is supported by noise.

The bottom-left panels in Figure 19 show entropy-based comparisons using permutation entropy (PE) and Shannon and Kolmogorov–Sinai (KS) comparisons. No matter the noise the Shannon entropy remains at its maximum, signaling a constant information content. PE is also responsive to short-time variations, as shown by the formation of localized spikes under stochastic forcing (Figure 19b,c), although in the deterministic situation PE is stable (Figure 19a) [47]). The dynamical richness of the system allows it to avoid high-dimensional chaos fully, as witnessed by the minimal KS entropy. Analysis of entropy indicates the existence of a great global informational structure in a broad sense, and noise makes the world vary at the local level without affecting the global processes.

The lower-right panels of Figure 19 (phase) show the time-varying permutation entropy (PE), which is computed with a moving window of size 200 to take into consideration localized complexity variations. These curves present the way stochasticity affects the temporal dynamics. Figure 19a shows quasi-periodic or lightly chaotic behavior of PE, which remains flat and stable. Short spikes and drops in Figure 19b denote the transition between uneven and noisy sounds. The repeating patterns of entropy, as shown in Figure 19c, can be seen to be separable into periodic patterns of noise that interrupt the systemic oscillations. With everything said and done, PE evolution shows the role played by randomness in the introduction of uncertainty, as well as the amplification of activity in otherwise smooth dynamics.

Applying quantitative tools to the quartic oscillator system (10), it is possible to see that the random noise and predictable dynamics both play a role in the system motion. Increased values of the Lyapunov exponent and entropy signify that noise increases the sensitivity and complexity of the system; however, the shape of attractor and overall wave-like forms remain constant. Permutation entropy is localized, yet the system is ordered and is confined. This is an example of why a combination of Lyapunov and entropy measures is important, because noise brings complexity to stability.

The simulations were executed in **Python 3.10** with the help of **NumPy**, **SciPy**, **Matplotlib**, and **tqdm**. Deterministic trajectories were solved using odeint (atol = $1\times 10^{-8}$, rtol = $1\times 10^{-6}$), time step = **0.01**, total span = 500, and initial state = [0.5,0.0]. Jacobian analysis of the Lyapunov is possible, and phase space, recurrence, and entropy are measures of deterministic dynamics. Stochastic simulations: Gaussian white noise with standard deviation = −s(sigma)eta (Delta) was included that was produced by:

Stochastic simulations incorporated Gaussian white noise with standard deviation $\sigma \sqrt{\Delta \eta }$, generated via scipy.stats.norm.rvs. Integration followed the Euler–Maruyama scheme used in the Ito sense, and different random seeds were used on a per-run basis. Averaging of five realizations produced credible measures of Lyapunov exponents and entropy. The smaller time steps were used to check the numerical stability of the convergence tests, and all the results, such as entropy, Lyapunov spectra, and phase-space trajectories, were stored and displayed in standardized forms.

### 3.9. Bifurcation Diagram and Lyapunov Spectrum Analysis

**Figure 20:**
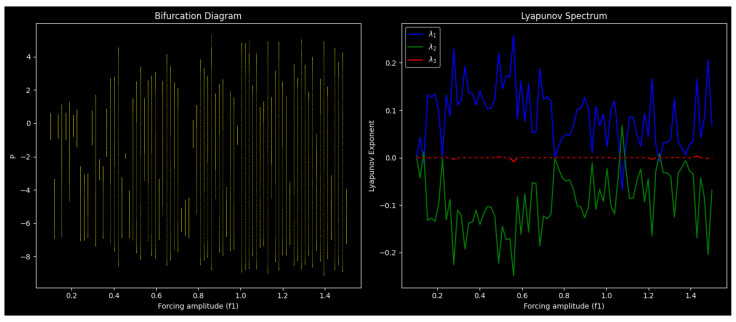
Time evolution of the largest Lyapunov exponent, converging to a positive value that indicates chaos.

The greatest exponent is positive, indicating chaotic divergence, whereas the Lyapunov exponent is indicative of the beginning circumstances of nonlinear systems. The largest Lyapunov exponent is used to measure the stability and chaos of simulated time series [48]. The bifurcation diagram in Figure 20 (on the left side) and the Lyapunov spectrum in Figure 20 (on the right side) of system (9) are obtained by taking *P* and *Z* as the second-order oscillators and *M* as the phase variable that periodically provides forcing.

The bifurcation pattern of *P* vs. forcing intensity $f_{1}\;\mathrm{within}\left[0.1,1.5\right]$ is shown on the left side. The majority of slices create dense point clouds over P∈[−9,5], which indicates aperiodic, chaotic motion. Short periodic or quasi-periodic windows are indicated by narrow filament-like regions close to $f_{1}\approx 0.7$–0.8 and 1.0–1.1, which indicate transient stabilization. This demonstrates how chaos and order can coexist, since slight adjustments to the parameters result in momentary regularity. Non-stroboscopic sampling highlights smooth transitions and broadens periodic solutions.

The Lyapunov spectrum from the Benettin–QR method is displayed in the right panel. With near-zero dips that line up with periodic windows and peaks near $f_{1}\approx 0.5$–0.6, the largest exponent $\lambda_{1}$ (blue) is primarily positive, confirming chaos interspersed with regular motion. Contraction is indicated by the second exponent $\lambda_{2}$ (green), while the neutral phase variable *M* is reflected by $\lambda_{3}$ (red), which hovers around zero. In periodically driven systems, strange attractors typically have this (+,−,0) structure.

Trajectory divergence is depicted in Figure 20, where the largest Lyapunov exponent $\lambda_{\max}$ is given by the slope of the first linear segment. Chaos is indicated by positive slopes, whereas periodic or stable motion is implied by zero or negative slopes. Lyapunov analysis and combined bifurcation show primarily chaotic dynamics with islands of order ($\lambda_{1}\;\to \;0$), while $\lambda_{3}\;\approx \;0$ represents the neutral phase variable *M* and maps dynamical regimes as $f_{1}$ changes.

The simulations were implemented in **Python 3.x** using the **SciPy** library’s solve_ivp function for numerical integration, employing the **Runge–Kutta** method with adaptive step size and high precision tolerances (rtol = $1\times 10^{-8}$ and atol = $1\times 10^{-8}$). The **NumPy** and **Matplotlib** libraries were used for numerical computation and visualization, respectively. The Lyapunov exponents were computed using the **Benettin algorithm** with a time step of **0.1** and total integration time of **50 s**.

Making sure that the Lyapunov spectra are numerically stable and repeatable, convergence experiments involving various integration tolerances and step sizes were performed prior to large-scale simulations. Stochastic extensions were generated using convergence tests, with white noise created by default with the function numpy.random.normal, and with the Euler–Maryama method the stochastic differential equations were integrated in the so-called Ito sense. Ensemble averaging was applied to minimize statistical and transitory bias to a number of initial conditions and distinct noise realizations. The implementation was checked against well-known chaotic systems to ensure the stability and repeatability of Lyapunov exponents at both deterministic and stochastic regimes.

#### Numerical Consistency Analysis of the Stochastic Quartic Oscillator

**Figure 21:**
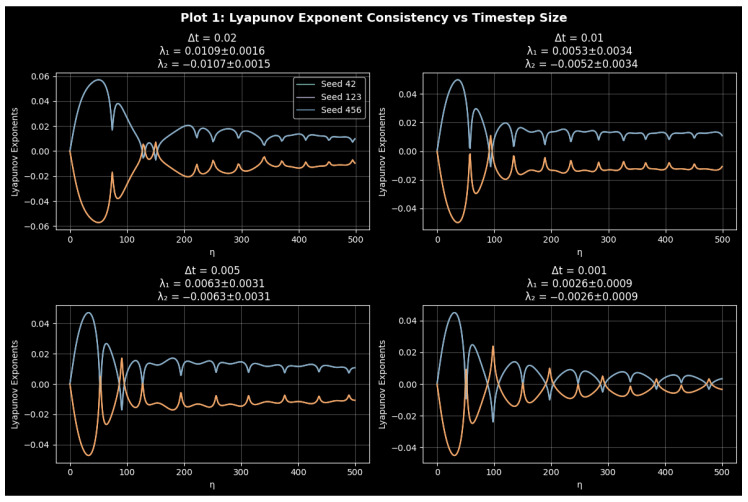
Time step sensitivity of Lyapunov exponents. Convergence of Lyapunov exponents for the stochastic quartic oscillator under time step refinement (Δη=0.02,0.01,0.005,0.001). The leading exponents remain consistent across resolutions, confirming that chaotic or quasi-periodic behavior is not a numerical artifact but an intrinsic feature of the dynamics.

To confirm that the reported dynamical signatures originate from the intrinsic physics of the system rather than from numerical artifacts, a detailed numerical consistency assessment was performed on the perturbed and noisy dynamical systems defined in Equations (8)–(10). The tests include time step refinement, random-seed variation, and deterministic–stochastic comparisons. This sensitivity analysis determines how sensitive the Lyapunov exponents of system (10) are to the size of the numerical integration of step Δη as shown in Figure 21. An Euler–Maruyama scheme of the system was implemented with enough scheme sizes of Δη=0.02,0.01,0.005, and 0.001, with additive Gaussian noise added to the $Z^{\prime }$–equation, i.e., $\sigma \sqrt{\Delta \eta }\;N\left(0,1\right)$. Ensemble variability was ensured with multiple random seeds.

The calculated Lyapunov traces $\left(\lambda_{1},\lambda_{2}\right)$ approach a steady point after transient phases, and their steady values are almost identical within numerical errors. The preservation of exponent ordering and sign structure confirms that the weakly chaotic or quasi-periodic behavior is not a spurious effect of discretization. Hence, the finite-time Lyapunov spectra of both the perturbed and noisy models are numerically stable under time step refinement.

**Figure 22:**
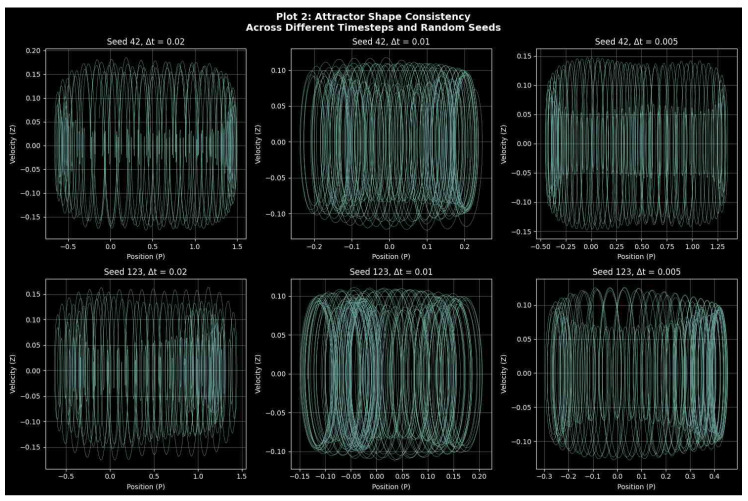
Attractor geometry under numerical discretization. Phase-space reconstruction of the stochastic quartic oscillator for multiple time steps, showing preserved global structure and invariant topological features. The attractor envelope, folding symmetry, and manifold continuity remain unchanged, confirming robustness to time step and seed variations.

Analysis of the attractor geometry under numerical discretizations is determine through Figure 22. Here we examine whether the phase-space topology of the extended system (9), represented by the coordinates (P,Z,M), remains invariant when Δη and the random-seed initialization are varied. Attractor reconstructions reveal nearly identical global envelopes, filament structures, and folding patterns across all tested step sizes and seeds.

Smaller time steps yield smoother trajectories, while stochastic realizations introduce only local blurring around deterministic manifolds. No qualitative deformation, collapse, or bifurcation into a new attractor is observed. This invariance demonstrates that the observed multistability and strange attractor morphologies are genuine features of the dynamical equations and not numerical artefacts arising from discretization or noise sampling.

**Figure 23:**
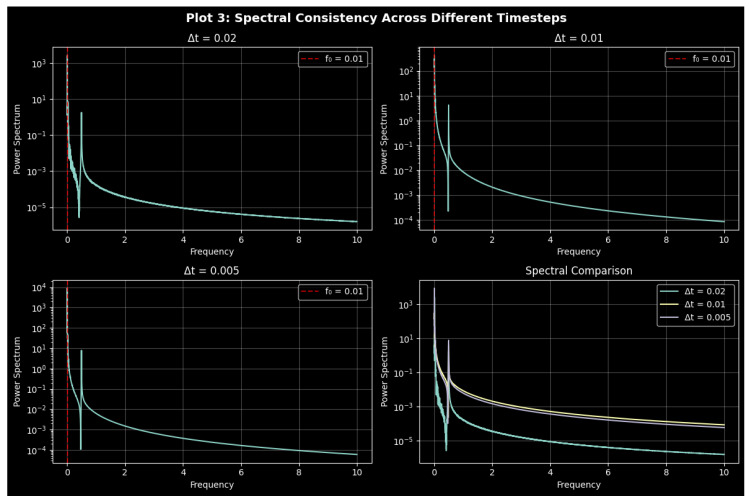
Spectral stability across integration parameters. Power spectral density (PSD) comparison for varying time steps. The main oscillatory modes and energy distribution remain identical, with only minor high-frequency aliasing at coarser resolutions. Dominant frequencies are preserved, indicating stable frequency-domain behavior under time step refinement.

We analyzed the spectral stability of the system across different integration parameters through Figure 23. The analysis is performed on the perturbed noisy quartic oscillator system (10). In order to confirm that the dominant timescales of an oscillator are maintained during numerical refinements, the power spectral density (PSD) of P(η) was calculated for all refinements of the numerical solutions of the stochastic differential equations in the Ito sense. Each spectrum exhibits the same principal low-frequency peaks associated with the forcing frequency *q* and its harmonics, along with a broadband decay reflecting the chaotic components of the motion.

Only the coarsest discretization (Δη=0.02) introduces minor high-frequency distortions due to aliasing, whereas refined time steps yield overlapping spectra with consistent energy distributions. The preservation of dominant frequencies and spectral envelopes confirms that the stochastic quartic oscillator’s oscillatory dynamics are numerically stable and not affected by integration parameter choices.

**Figure 24:**
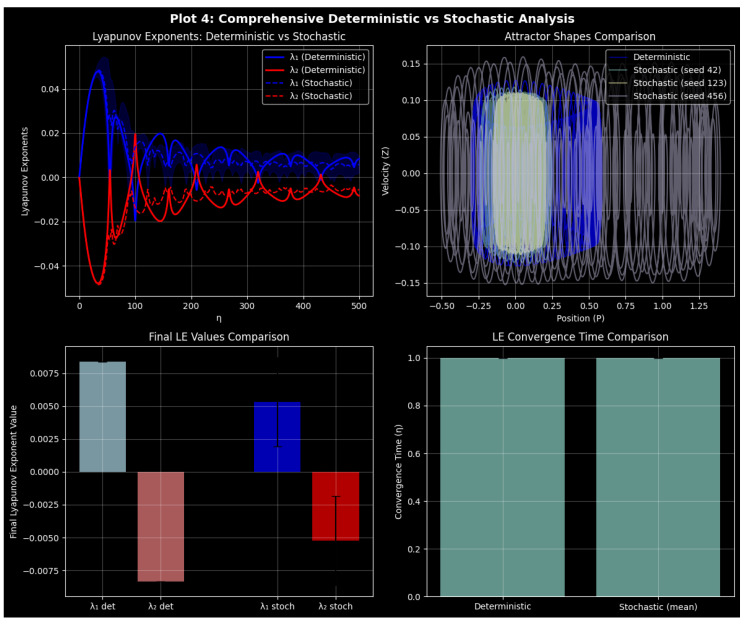
Deterministic–stochastic comparative dynamics. Comparison between deterministic and noise-driven realizations of the quartic oscillator. Stochastic perturbations slightly broaden attractor envelopes and introduce finite-time Lyapunov fluctuations, yet the qualitative dynamical invariants—dimensionality, spectral modes, and sign structure—remain consistent, confirming structural resilience under noise.

Comparative dynamics between deterministic and stochastic regimes are shown in Figure 24. This comparative test contrasts the deterministic system (8) with its noisy counterpart (10) to evaluate the role of stochastic perturbations. The deterministic trajectories produce compact, smooth attractors and steady Lyapunov convergence, while the stochastic integrations slightly broaden the attractor and introduce small-amplitude fluctuations in the finite-time exponents.

These variations do not affect the overall dynamical invariants, including spectral composition, the dimensionality of the attractor, and the structure of the Lyapunov spectrum. Instead of introducing new attractors or changing the qualitative regime, the extra noise primarily diffuses on already existing manifolds. This shows that the stochastic extension is what maintains the fundamental nonlinear dynamics of the quartic oscillator.

The quartic oscillator has a high numerical consistency type in all diagnostic examinations. The robustness of the Lyapunov spectra, geometries of the attractors, and spectral profiles are not influenced by stochastic perturbations, seed variation, and time step refinement. As such, the transitions in periodic, quasi-periodic, and chaotic regimes reported are not numerical aberrations but instead are natural events in dynamical processes.

The simulations were executed in **Python 3.x** using key scientific libraries, including **NumPy** for numerical operations, **SciPy** for numerical integration of the governing system via the odeint solver, and **Matplotlib** for visualization. The implementation also incorporated **SciPy.fft** for spectral analysis and **tqdm** for progress tracking, ensuring both computational efficiency and clarity during long simulations. The integration was performed over the interval η∈[0,500] using varying time steps (Δη=0.02 to 0.001) to examine time step sensitivity and numerical convergence.

In order to confirm that the dominant timescales of an oscillator are maintained during numerical refinements, the  scipy.stats.norm.rvs and Euler–Maruyama method were used to calculate the numerical solutions of the stochastic differential equations in the Ito sense for all refinements. To verify fault resilience to the discretization error, the geometry of attractors and spectral density were analyzed for both deterministic and stochastic regimes. The calculation of Lyapunov exponents was performed on a modified Benettin algorithm re-orthogonalized with QR. Consistency between deterministic and stochastic regimes was evaluated using comparative measures such as convergence rate, spectral peaks, and attractor topology and ensured the stochastic simulations are robust, reproducible, and numerically stable in response to seeds, time steps, and noise realizations.

Moreover, all the parameter windows that exhibited regular or almost-regular behavior were studied closely to provide the classification of the observed “order windows” in the bifurcation diagrams and Lyapunov spectra. Periodic windows were identified in sharp peaks in the power spectral density, disappearing maximal Lyapunov exponents $\lambda_{\max}\approx 0$), and discrete, well-separated points in Poincaré sections. Quasi-periodic windows were characterized by closed or toroidal phase-space structures, multiple, incommensurate spectral peaks, and small yet non-zero maximal Lyapunov exponents, indicating confined and non-repeating motion. Sensitive dependence on initial conditions, broadband spectra, and positive maximal Lyapunov exponents both pointed in favor of the existence of chaotic intervals.

These classifications were tabulated, and bifurcation diagrams were created systematically so as to map out in detail the periodic, quasi-periodic, and chaotic regimes between the ranges of parameters explored. In particular, narrow “order windows” embedded within otherwise chaotic regions were highlighted, showing transitions from high-dimensional chaos to periodic or quasi-periodic dynamics and back. This improved analysis makes it possible to obtian a more precise view of the global dynamical structure of the system and how changes in different parameters affect stability, multistability, and stochastic robustness.

### 3.10. Variance-Based Sensitivity Analysis of the Stochastic SIdV Equation

The Sobol approach was used to assess the influence of modifications to parameters on stochastic SIdV dynamics as part of a variance-based global sensitivity assessment. This study demonstrates how slight changes in parameters lead to shifts from order to chaos by analyzing the output variance of observables such amplitude variance, the Lyapunov exponent, and spectral entropy. This makes it possible to quantify the robustness and complexity of the system.

**Figure 25:**
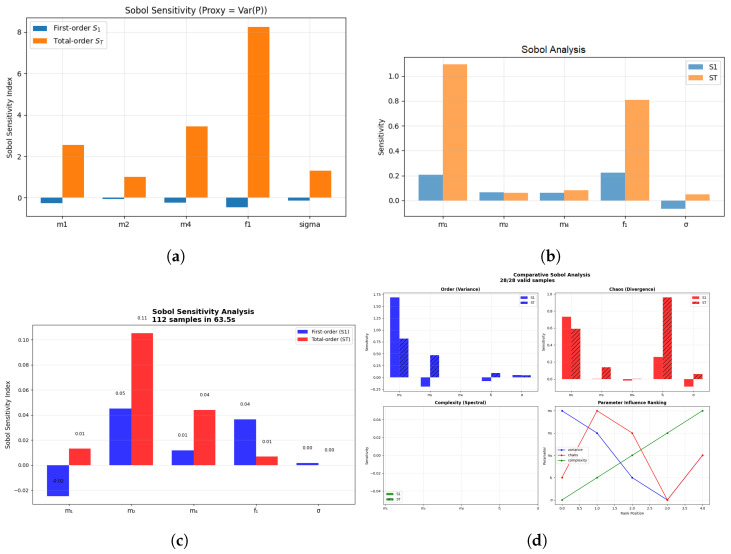
Comprehensive Sobol-based variance sensitivity analysis of the stochastic SIdV system. (**a**) Variance sensitivity, (**b**) Lyapunov sensitivity, (**c**) spectral entropy sensitivity, and (**d**) overall parameter ranking by total-order indices.

Figure 25a–d display Sobol sensitivity data based on variance. **(a) Variance Sensitivity:** Cubic nonlinearity $m_{4}$ along with forcing amplitude $f_{1}$ show the largest Sobol indices, influencing amplitude growth, whereas linear stiffness $m_{1}$ provides significant stabilization and $m_{2}$ and σ have insignificant effects. **(b) Lyapunov Sensitivity:** The Lyapunov proxy becomes most sensitive to $m_{4}$ and $f_{1}$, supporting nonlinear–forcing coupling as the main cause of chaos. $m_{1}$ suppresses divergence, and σ slightly increases instability. **(c) Spectral Entropy Sensitivity:** While $m_{4}$ and $f_{1}$ once again dominate, expanding spectra and increasing complexity, σ maintains weakly nonlinear environments but boosts complexity under strong nonlinearity. **(d) Parameter Ranking:** A consistent hierarchy $m_{4},f_{1}\gg m_{1}>\sigma >m_{2}$ appears, suggesting that in the stochastic SIdV system, nonlinear–forcing interactions largely govern robustness and chaos, with noise altering resilience and stiffness enhancing stability. Essentially, by identifying critical factors influencing chaotic transitions, the Sobol approach improves interpretability, reproducibility, and overall parameter modification for experimental validation. The "Complexity (Spectral)" subplot presently displays blank bars, suggesting that the complexity measure could not be calculated for any of the valid samples utilized in this study. This result does not indicate a general failure of the procedure; rather, it reflects the special characteristics of the samples examined here. If alternative sample values are utilized, the spectral complexity accessible may produce non-zero results, indicating sensitivity to the data underneath.

By demonstrating that stiffness ($m_{1}$) stabilizes, noise (σ) modifies robustness, and cubic nonlinearity ($m_{4}$) and forcing ($f_{1}$) drive chaos–order transitions, Sobol analysis offers quantitative insight into stability and guides parameter selection (see Table 4).

Simulations were performed in **Python 3.x** using **NumPy** for computation, **SciPy** (odeint) for integration, and **Matplotlib** for visualization. **SciPy.fft** enabled spectral analysis and **tqdm** progress tracking. Integration over η∈[0,500] with time steps Δη=0.02–0.001 tested time step sensitivity and convergence.

Stochastic forcing was described as Gaussian white noise using scipy.stats.norm.rvs and integrated in the Itô sense utilizing the Euler–Maruyama technique. Ensemble reliability was guaranteed by several Monte Carlo runs using separate seeds. In order to verify numerical stability, convergence, and robustness to noise and discretization, Lyapunov exponents were calculated using a modified Benettin algorithm, and attractor geometry and spectra were compared across deterministic and stochastic regimes.

### 3.11. Analysis of the Perturbed Hamiltonian System

This study investigates a one-degree-of-freedom nonlinear Hamiltonian system with canonical coordinates (P,Z), subject to periodic forcing. The aim is to compare the unperturbed (conservative) case with the perturbed dynamics and assess energy evolution. The Hamiltonian is

(12) $$H\left(P,Z,\eta \right)=\frac{1}{2}Z^{2}+V\left(P\right)-f_{1}P\cos\left(\omega \eta \right),$$

where the potential is

(13) $$V\left(P\right)=m_{4}P^{4}+m_{2}P^{2}+m_{1}P,$$

and $f_{1}$ and ω in Equation (12) are the forcing amplitude and frequency. The external term $f_{1}\cos\left(\omega \eta \right)$ breaks time invariance, enabling energy exchange with the driving field.

Numerical integration is carried out using solve_ivp with initial conditions P(0)=0.1 and Z(0)=0.0 over η∈[0,50], employing a tolerance of $10^{-6}$. Large *P* values are permitted to avoid overflow, and non-finite results are filtered prior to visualization.

**Figure 26:**
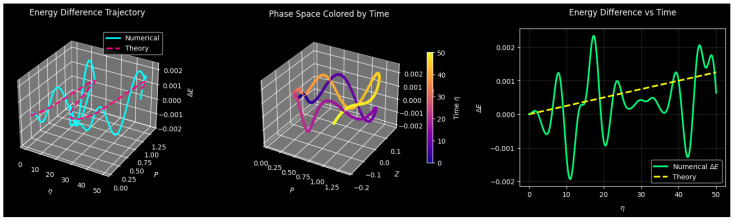
Three views of perturbed dynamics: (**Left**) 3D energy–time–position plot, (**Middle**) time-colored phase-space trajectory, and (**Right**) energy difference showing oscillations and secular growth.

Three complementary panels illustrate the simulations in Figure 26. The left panel shows the energy trajectory in 3D, where the evolution of ΔE in (η,P) indicates that the numerical solution (cyan) oscillates around the theoretical prediction (magenta dashed), confirming perturbation theory at early times and revealing nonlinear deviations later. The nonlinear aberrations of the secular energy growth of the perturbed system can be exhibited through distorted (P,Z) curves and the gradual increase in the energy in the perturbed system. Previous consensus confirms this approximation, but the reasons are later resonances, along with higher-order effects. Precision is performed numerically through high attention to solver parameters and the graphics description. On the whole, the results emphasize the usefulness of nonlinear analysis methods, such as phase-space reconstruction and correlation dimension calculation [33], as well as confirm and extend the perturbative predictions and demonstrate how weak periodic perturbations lead to the deformation of structures and energy drift [37].

All simulations were performed in Python using NumPy and SciPy for integration (solve_ivp, rtol = $1\times 10^{-6}$) and Matplotlib for 3D trajectory and phase-space visualization.

Prior to steady-state analysis, simulations employed near-equilibrium initial conditions with transients eliminated. Time step refinement was used to confirm convergence. Gaussian white noise (numpy.random.normal) summed in the Itô sense using the Euler–Maruyama technique was used in stochastic runs. For statistical reliability, ensemble averaging over separate realizations was used. The framework guaranteed long-term integration that was accurate, effective, and steady.

## 4. Comparative Context with Previous Studies

To contextualize these findings, we add stochastic forcing alongside soliton overlap dynamics to recent deterministic phase-space studies in *Physica D*.

In [49,50], phase-space studies of deterministic systems, including photon–magnon along with magnetoelectric couplings, focused on equilibria, Hopf bifurcations, and particularly deterministic coupling effects. In contrast, this work investigates noise-induced perturbations, order–chaos transitions, and multistability in a stochastic SIdV system. By applying static bifurcation investigations to dynamic, noise-driven attractor development and evaluating soliton robustness through overlap analysis across deterministic and stochastic regimes, it offers a more realistic perspective on nonlinear wave behavior.

## 5. Conclusions and Future Directions

In order to determine the combination of deterministic and stochastic variations, we studied the nonlinear dynamics of the generalized stochastic intermediate dispersive velocity (SIdV) equation in detail. The causes of the complexity of the system are the natural nonlinearity making it chaotic and the external stochasticity changing it, either enhancing or reducing dynamics as a result of noise characteristics.

Numerical integration, phase-space reconstruction, Lyapunov exponents, power spectra, recurrence quantification, entropy measurements, correlation dimension, return maps, basin stability, and bifurcation analysis were used in the analysis of the transformed ordinary differential equations. The results revealed many dynamics, including weak chaos, quasi-periodicity, and full stochastic behavior.

The nature of noise greatly affected the system because the white noise kept the core dynamics intact, whereas the Brownian and colored noise highlighted chaos and alleviated attractors structures. Soliton overlap and basin stability were used to test the strength of the coherent wave structures, fractal boundaries, and multistability.

Soliton resilience and noise-dependent multistability give us data on stabilizing or destabilizing wave patterns that are more appropriate in energy harvesting and noise-regulated optical devices, which are useful for prediction structures in safe communication and signal modulation.

This paper demonstrates the interaction between nonlinear coupling and stochastic excitation to stabilize and control the complexity of the system. Consistency tests ensure that the transitions are physical and not numerical, and autocorrelation is a memoryless chaotic diffusive test.

Chaos, stochastic forcing, small order windows, multilistability, bifurcation diagrams, and Lyapunov spectra have been systematically studied to reveal periodic and quasi-periodic and chaotic regions.

Measurements of the effects of nonlinearities, stiffness, forcing, and noise on amplitude variance, Lyapunov divergence, and spectral entropy by Sobol studies demonstrate that noise corrects robustness, whereas nonlinear–forcing interactions lead to chaos. Combined with the concepts of bifurcation and Lyapunov analysis, this offers an official account to predict the stochastic soliton dynamics.

In this way, our work builds upon our expertise in stochastic soliton dynamics, offers a means of evaluating resilience, sensitivity, and predictability, and, hence, contributes to the experimental validation, noise control, and resilient wave-based applications in energy, optics, fluids, and signal processing.

### Future Directions

Future research ought to be on experimentally confirming the dynamics of fluid, optical, or electrical systems when real noise sources can be introduced and controlled.

The impacts of multiplicative noise and parameter uncertainty on soliton stability should be examined in further studies with the help of the perturbation-based techniques, i.e., Riccati expansions, the modified Kudryashkov approach, or stochastic averaging. The emergence of new regimes of synchronization, noise-induced order–chaos transitions, and stochastic bifurcations could be observed after applying Monte Carlo and Polynomial Chaos Expansion studies to coupled or higher-dimensional SIdV systems.

Another interesting strategy is stabilizing, which involves the development of control, including adaptive control, delayed control, or feedback control.

Together with sensitivity and bifurcation analyses, data-based neural operator and DMD methods, as well as SINDy, may provide simplified and noise-resistant models that help to discover key parameters and guide controls design.

Large deviation principles, stochastic bifurcation theory, and complexity metrics like spectral entropy, multiscale entropy, and persistent homology can be used to quantify the stochastic effects on soliton resilience and stability and analyze noise-induced transitions. To improve parameter estimates and predictability even more, information-theoretic and Bayesian techniques can be applied.

Higher-order nonlinearities, coupled SIdV systems, and non-Gaussian noise (like Lévy or Poisson processes) can expand the framework’s applicability to realistic wave systems for precise stochastic soliton forecasting and management in energy, optical, and signal processing applications.

These initiatives seek to bridge theory and applications by converting recent discoveries into workable methods for managing stochastic nonlinear waves.

## Figures and Tables

**Table 1:** Parameter sets, integration step sizes, and time spans used in the simulations.

Figure	Parameters	Step Size	Time Span
Figure 9	[−0.05,0.5,−0.8,0.9,0.1]	0.01	η∈[0,500]
Figure 10	[−0.05,0.5,−0.8,0.9,0.1]	0.005	η∈[0,1000]
Figure 11	[−0.04,0.6,−0.85,1.0,0.1]	0.005	η∈[0,1000]

**Table 2:** Comparison of the system’s response under different noise types.

Aspect	White Noise	Brownian Noise	Colored Noise
Amplitude	0.1	0.05	0.05
Power Spectrum	Smooth decay, low variability	High low-frequency power, high variability	Clean decay, moderate variability
Peak Frequency	Sharp and centered	Bimodal, unstable	Sharp and centered
Oscillations	Robust and preserved	Often disrupted	Preserved with mild dispersion
Stability	Stable, oscillations persist	Unstable, drift and incoherence	Partially stable, moderate variability

**Table 3:** Quantitative measures contrasting dynamical properties.

Metric	Original	Surrogates (Mean ± std)
Correlation dimension	2.34	1.02 ± 0.15
Recurrence rate	0.41	0.18 ± 0.03
$\tau_{\mathrm{decorrelation}}$	>1000	142 ± 28

**Table 4:** Summary of Sobol sensitivity results across subplots (a)–(d).

Plot	Model Complexity	Key Parameters	Physical Role	Computational Focus
(a)	Full nonlinear + stochastic	$f_{1}$, $m_{4}$	Chaos resonance	Balanced realism
(b)	Linear simplified	$m_{1}$	Stability amplitude	Maximum speed
(c)	Nonlinear truncated	$f_{1}$, $m_{4}$, $m_{2}$	Nonlinear amplification	Fast with realism
(d)	Multi-metric stochastic	$m_{1}$, $m_{4}$, $f_{1}$, σ	Order–chaos transitions	Multi-observable insight

## Data Availability

All data generated or analyzed during this study are included in this published article.
